# New Insights into Dietary L-Glutamate and L-Aspartate Modulation of Hematology, Immune Responses, and Metabolite Profiles in Enterotoxigenic *Escherichia coli* Challenged Piglets

**DOI:** 10.3390/metabo16040247

**Published:** 2026-04-04

**Authors:** Supatirada Wongchanla, Sangwoo Park, Shuhan Sun, Peng Ji, Yanhong Liu

**Affiliations:** 1 Department of Animal Science, University of California, Davis, CA 95616, USA; swongchanla@ucdavis.edu (S.W.); wamsun@ucdavis.edu (S.S.); 2 Division of Animal Bioscience and Integrated Biotechnology, Gyeongsang National University, Jinju 52828, Republic of Korea; swppark@gnu.ac.kr; 3 Department of Nutrition, University of California, Davis, CA 95616, USA; penji@ucdavis.edu

**Keywords:** *Escherichia coli*, immune response, L-aspartate, L-glutamate, metabolomics, weaned piglets

## Abstract

Background/Objectives: L-glutamate (Glu) and L-aspartate (Asp) are key intermediates in nitrogen metabolism and tricarboxylic acid cycle activity, linking intestinal energy metabolism with immune function. This study investigated how dietary Glu and Asp supplementation modulates immune responses and metabolic reprogramming in weaned pigs challenged with F18 enterotoxigenic *Escherichia coli* (ETEC). Methods: Forty-nine piglets (24 d old; 8.18 ± 1.54 kg body weight) were randomly assigned to seven treatments (*n* = 7/treatment): unchallenged control (NC), ETEC-challenged control (PC), 1% or 2% Glu, 1% or 2% Asp, and an antibiotic control. The experiment was conducted from d −7 to d 14 post-inoculation (PI). Hematological indices, serum biomarkers, intestinal cytokine gene expression, and untargeted metabolomic profiling of serum, ileal mucosa, and ileal digesta were evaluated. Results: On day 2 PI, 1% Glu reduced the neutrophil-to-lymphocyte ratio, whereas 2% Asp showed an elevated ratio. Supplementation of 1% Asp increased serum total protein on d 2 and d 5 PI. On d 14 PI, 1% Glu enhanced jejunal *IL-17A* and *IL-22* expression, while 2% Asp reduced jejunal *IL-6* expression compared with PC. Ileal *IL-12* expression increased with 1% Glu and 2% Asp, whereas jejunal *IL-12* expression decreased with 2% Glu and 2% Asp. Untargeted metabolomics revealed distinct treatment-dependent separations. Differential metabolite profiling and pathway enrichment analyses demonstrated coordinated alterations in amino acid metabolism, purine metabolism, lipid metabolism, and energy-related pathways across serum and intestinal compartments. Conclusions: Collectively, Glu and Asp supplementation reshaped host metabolic networks during ETEC challenge, indicating their roles in modulating metabolic adaptation and intestinal immune–metabolic crosstalk under enteric stress.

## 1. Introduction

The weaning transition represents a critical period in swine production, during which piglets are exposed to multiple environmental, dietary, and physiological stressors. The immature immune system of post-weaning piglets increases their susceptibility to pathogenic infections, particularly ETEC, a major etiological agent of post-weaning diarrhea (PWD) [1,2]. ETEC colonizes the small intestine through fimbrial adhesins (e.g., F4 or F18), which facilitate attachment to enterocytes [3]. Once adhered, the bacteria secrete enterotoxins, heat-labile (LT) or heat-stable (STa, STb), that disrupt electrolytes and fluid homeostasis by promoting excessive chloride and water secretion into the intestinal lumen, resulting in watery diarrhea, dehydration, and nutrient malabsorption [4,5]. The infection also triggers local and systemic immune responses, characterized by proinflammatory cytokine production and mucosal inflammation, further impairing intestinal barrier functions [5]. In parallel, ETEC infection induces metabolic alterations in the host, including increased energy demand, reduced nutrient assimilation, and shifts in amino acid and lipid metabolism [6]. This condition contributes to poor growth, increased morbidity and mortality, and substantial economic losses in swine production.

Although the use of antimicrobial agents has been effective in managing PWD, growing concerns over antimicrobial resistance have prompted global restrictions on the use of antibiotics for growth promotion in livestock, including swine farming [7,8,9]. Consequently, the swine industry has shifted its focus toward alternative strategies such as enhanced biosecurity, vaccination, genetic selection, and nutritional interventions to support gut health and resilience during the weaning period.

Among nutritional strategies, supplementation with functional amino acids has gained attention as a means to mitigate weaning stress and support intestinal health. L-glutamate (Glu) and L-aspartate (Asp) are considered functional amino acids that play multifaceted roles in the intracellular metabolism of mammalian enterocytes [10,11,12]. They serve as major energy sources for intestinal cells by donating their carbon skeletons to the tricarboxylic acid (TCA) cycle, thereby generating adenosine triphosphate (ATP) to support various cellular processes [13,14]. Additionally, they function as intermediates in amino acid biosynthesis and contribute to nucleotide synthesis [15]. Glu and Asp may also enhance cellular antioxidant systems and modulate immune responses [16,17,18]. Previous reports have demonstrated that dietary supplementation with Glu or Asp in weaned pigs can enhance growth performance, reduce oxidative stress, strengthen intestinal barrier function, and modulate gut immune responses [11,17,18]. Although Glu and Asp are traditionally classified as non-essential amino acids and naturally abundant in typical animal feed ingredients, the low feed intake commonly observed during weaning can lead to nutrient insufficiencies [19]. Combined with the heightened metabolic demands of modern, fast-growing pig breeds, this suggested that Glu and Asp may become conditionally essential nutrients to support optimal growth and development under weaning stress [11,12,20].

In the present pilot study, a nutritional approach was investigated to mitigate PWD by supplementing weaned pig diets with L-glutamate (Glu) and L-aspartate (Asp) [21]. Given their roles as functional amino acids involved in key intestinal cellular processes, this study aimed to explore the in vivo effects of Glu and Asp on immune responses and metabolite profiles, characterizing both systemic and intestinal responses in weaned pigs challenged with ETEC. We hypothesized that Glu and Asp may alleviate intestinal inflammation through modulation of immune and metabolic pathways, thereby supporting host defenses against ETEC and reducing reliance on antibiotic interventions in swine production.

## 2. Materials and Methods

### 2.1. Animals, Housing, Experimental Design, and Diet

The protocol for this experiment was reviewed and approved by the Institutional Animal Care and Use Committee (IACUC #21875) of the University of California, Davis (UC Davis). A total of 49 weanling piglets (24 d old; 8.18 ± 1.54 kg body weight (BW)) were used in this experiment. The 6 sows and piglets used in this experiment were not given *E. coli* vaccinations, antibiotic injections, or antibiotics in creep feed. All piglets utilized in this experiment had not previously been infected with F18 ETEC. After weaning, pigs were housed in individual pens (0.61 m × 1.22 m) at the Cole facility at UC Davis. Pigs were contained in individual pens for 21 d, including a 7 d adaptation period and 14 d following the first ETEC inoculation. All pigs were given free access to the diet and water. Animal units were provided with ventilators and heaters to create the ideal thermoneutral zone for nursery piglets. Light was provided for 12 h a day from 07:30 to 19:30. The experimental design, housing, management, and ETEC inoculation procedures followed our previous study [21].

Piglets were randomly allocated to one of seven treatments, with seven piglets per treatment, using a randomized complete block design with weight and sex as blocks and the individual pig as the experimental unit. The treatments included: (1) negative control (NC), basal diet without ETEC challenge, (2) positive control (PC), basal diet with ETEC challenge, (3) basal diet supplemented with 1% Glu with ETEC challenge, (4) basal diet supplemented with 2% Glu with ETEC challenge, (5) basal diet supplemented with 1% Asp with ETEC challenge, (6) basal diet supplemented with 2% Asp with ETEC challenge, and (7) basal diet supplemented with carbadox (Car) at 50 mg/kg and with ETEC challenge. Both Glu (Kosher/Orthodox Union, New York, NY, USA) and Asp (Anhui Sealong Biotechnology, Bengbu, Anhui, China) had a purity of ≥99% and were supplemented in free amino acid powder form to ensure accurate dosing and consistent bioavailability. The low inclusion level (1%) was intended to meet estimated nutritional requirements under weaning stress, while the high inclusion level (2%) was used to assess whether greater supplementation would elicit enhanced physiological responses or reveal a dose-dependent effect. These levels were chosen to help identify the optimal dosage for improving disease resistance in piglets. All diets met current nutrient requirement estimates for nursery pigs (Supplementary Tables S1 and S2) [22] and were fed in a 2-phase feeding program, with weeks 1 and 2 serving as phase 1 and weeks 3 as phase 2.

All pigs in the ETEC challenged treatments were orally inoculated with F18 ETEC (10^10^ CFU per 3 mL dosage in phosphate-buffered saline) for 3 consecutive days from d 0 before inoculation to d 2 PI. The strain produces heat-labile (LT) and heat-stable enterotoxins (STa and STb), and the inoculation dose has been shown to induce mild diarrhea in our previous study [23,24]. All piglets were confirmed to be susceptible to F18 ETEC using a genotyping procedure [21].

### 2.2. Sample Collection Procedures

The experiment terminated with the euthanasia of all pigs on d 14 PI. Pigs were anesthetized intramuscularly with a 1 mL mixture of 100 mg Telazol, 50 mg ketamine, and 50 mg Xylazine (2:1:1). Following anesthesia, each pig was euthanized with an intracardiac injection of 78 mg Fatal-Plus solution (sodium pentobarbital, MWI Animal Health, Visalia, CA, USA) per 1 kg of body weight. Ileal digesta samples were collected in snap-frozen tubes immediately after euthanasia, frozen in liquid nitrogen, and stored at −80 °C until analysis. Approximately 10 cm of the intestine was dissected longitudinally and gently washed with PBS to remove luminal contents for intestinal mucosa collection. Mucosa from the middle of the jejunum and ileum was scraped with a glass slide, promptly frozen in liquid nitrogen, and stored for gene expression analysis. Blood samples from all pigs were drawn from the jugular vein, with or without EDTA to collect whole blood and serum samples, respectively, on d 0 before inoculation, d 2, d 5, and d 14 PI.

### 2.3. Assessment of Complete Blood Count and Serum Inflammatory Biomarkers

Total and differential blood cell counts were determined on whole blood samples by the Comparative Pathology Laboratory at UC Davis. The assay to optimally distinguish porcine blood was conducted using a multiparameter, automated programmed hematology analyzer (Drew/ERBA Scientific 950 FS Hematological Analyzer, Drew Scientific Inc., Miami, FL, USA). The concentrations of serum haptoglobin (Hp) (Aviva Systems Biology Corp., San Diego, CA, USA), serum tumor necrosis factor-alpha (TNF-α), and serum C-reactive protein (CRP) (R&D System Inc., Minneapolis, MN, USA) in serum samples were analyzed in duplicate by enzyme-linked immunosorbent assays (ELISA) according to the manufacturers’ instructions. The intensity of the color was measured at 450 nm with the correction wavelength set at 530 nm using a plate reader (BioTek Instruments, Inc., Winooski, VT, USA). The concentrations of each analyte in the tested samples were calculated based on a standard curve.

### 2.4. Quantitative Analysis of Intestinal Immune-Related Gene Expression

Reverse transcription quantitative polymerase chain reaction (RT-qPCR) was employed to assess gene expression in jejunal and ileal mucosa samples. Approximately 100 mg of each mucosa sample was homogenized with TRIzol reagent (Invitrogen; Thermo Fisher Scientific, Inc., Waltham, MA, USA). Subsequently, total ribonucleic acid (RNA) was extracted following the RNA extraction protocol recommendations provided by the reagent manufacturer. The High-Capacity Complementary Deoxyribonucleic acid (cDNA) Reverse Transcription Kit (Applied Biosystems; Thermo Fisher Scientific, Inc., Waltham, MA, USA) was used to generate cDNA from 1 μg of total RNA per sample in a 20 μL volume. To ensure RNA purity, absorbance ratios at 260/280 and 260/230 nm were measured with a Thermo Scientific NanoDrop 2000 Spectrophotometer (Thermo Scientific, Inc., Waltham, MA, USA). The mRNA abundance of genes related to intestinal immune responses in jejunal and ileal mucosa were analyzed, including tumor necrosis factor-alpha (*TNF-α*), interleukin-1 beta (*IL-1β*), interleukin-6 (*IL-6*), interferon-gamma (*IFN-γ*), interleukin-12 (*IL-12*), transforming growth factor-beta 1 (*TGF-β1*), interleukin-10 (*IL-10*), interleukin-17A (*IL-17A*), interleukin-22 (*IL-22*), and interleukin-23 (*IL-23*). Data normalization was achieved using 18S ribosomal RNA (*18S rRNA*) as a housekeeping gene, which demonstrated stable expression across all samples and conditions, validating its suitability for normalization. Primers were designed according to published literature and commercially produced by Integrated DNA Technologies in Coralville, IA, USA. Each primer pair was verified and optimized through standard curve analysis, with amplification efficiencies ranging from 90% to 110%, ensuring reliability and consistency in RT-qPCR performance (Supplementary Table S3). A no-template control (NTC) was included as a negative control to assess reagent contamination and the potential presence of genomic DNA. The analysis of relative gene quantification in comparison to the negative control was conducted using the 2^−ΔΔCT^ method. The RT-qPCR reaction conditions adhered to the protocols outlined in the published research [23].

### 2.5. Untargeted Metabolomics Analysis

Untargeted metabolomics analysis was conducted by the NIH West Coast Metabolomics Center at the University of California, Davis, using gas chromatography (Agilent 6890 gas chromatograph controlled using Leco ChromaTOF software version 2.32, Agilent Technologies, Santa Clara, CA, USA) coupled with time-of-flight mass spectrometry (GC/TOF-MS) (Leco Pegasus IV time-of-flight mass spectrometer controlled using Leco ChromaTOF software (version 3 2.32), LECO Corp., St. Joseph, MI, USA). Metabolite extraction was performed following procedures described previously [25]. Briefly, frozen serum from d 5 PI, ileal mucosa, and ileal digesta samples from d 14 PI (approximately 10 mg) were homogenized using a Retsch ball mill (Retsch, Newtown, PA, USA) for 30 s at 25 times/s. After homogenization, a prechilled (−20 °C) extraction solution (isopropanol/acetonitrile/water at the volume ratio 3:3:2, degassed with liquid nitrogen) was added at a volume of 1 mL/20 mg of sample. Samples were then vortexed and shaken for metabolite extraction. After centrifugation at 12,800× *g* for 2 min, the supernatant was collected and divided into two equal aliquots and concentrated at room temperature for 4 h in a cold-trap vacuum concentrator (Labconco Centrivap, Kansas City, MO, USA). To separate complex lipids and waxes, the residue was re-suspended in 500 µL of 50% aqueous acetonitrile and centrifuged at 12,800× *g* for 2 min. The resultant supernatant was collected and concentrated in the vacuum concentrator. Dried sample extracts were derivatized and mixed with internal retention index markers (fatty acid methyl esters with the chain length of C8 to C30). The samples were injected for GC/TOF analysis, and all samples were analyzed in a single batch. Data acquisition by mass spectrometry and mass calibration using FC43 (perfluorotributylamine) before starting analysis sequences. Metabolite identifications were performed based on the two parameters: (1) Retention index window ±2000 U (around ±2 s retention time deviation), and (2) Mass spectral similarity plus additional confidence criteria as detailed below (Data processing). A detailed methodology for data acquisition and metabolite identification was described in a previously published article by Fiehn et al. (2008) [25].

### 2.6. Metabolomics Data Processing and Analysis

The metabolomics data were analyzed using different modules of a web-based platform, MetaboAnalyst 6.0 (Xia Lab, McGill University, Montreal, QC, Canada; https://www.metaboanalyst.ca, accessed on 12 September 2024). Data were normalized using auto-scaling (mean-centered and divided by the standard deviation) to allow comparability among metabolites with different concentration ranges. Mass univariate analysis was performed using one-way ANOVA followed by Fisher’s least significant difference (LSD) test, with adjusted *p* ≤ 0.05 considered significant. Fold change analysis and *t*-tests were performed to assess the magnitude and significance of changes in individual metabolite levels. Statistical significance was determined using the false discovery rate (FDR) method with Benjamini–Hochberg correction. Given the exploratory nature of untargeted metabolomics and the large number of metabolites analyzed relative to the sample size, an FDR threshold of <0.2 was applied to balance false discovery control with sensitivity for detecting biologically relevant metabolic changes. Metabolites with an FDR < 0.2 and a fold change > 2.0 were considered upregulated, while those with a fold change < 1.0 were considered downregulated. Partial least squares discriminant analysis (PLS-DA) was conducted to identify discriminative metabolites among treatment groups. Variable Importance in Projection (VIP) scores were used to identify key discriminative metabolites, with VIP > 1 considered biologically relevant contributors to group separation. Pathway analysis and metabolite set enrichment analysis were performed on metabolites with a VIP score > 1. Pathways with a *p* ≤ 0.05, FDR < 0.2, and an impact value > 0.1 were considered significantly impacted.

### 2.7. Statistical Analysis

Data normality was assessed using the UNIVARIATE procedure of SAS (v9.4, SAS Inst. Inc., Cary, NC, USA). All data, except metabolomics data, were analyzed using the MIXED procedure of SAS. The statistical model included dietary treatment as a fixed effect and block (sex and initial body weight) as random effects, with the individual pig considered the experimental unit.

The model was expressed as

*Y*_ij_ = μ + *T*_i_ + *B*_j_ + ε_ij_,

where *Y*_ij_ is the dependent variable, μ is the overall mean, *T*_i_ is the fixed effect of treatment, *B*_j_ is the random effect of block, and ε_ij_ is the residual error.

For variables measured repeatedly over time, time and the treatment × time interaction were included as fixed effects, and pig was specified as the subject of repeated measures. Treatment means were estimated using the LSMEANS statement and separated using the PDIFF option with Tukey adjustment. Statistical significance and tendencies were declared at *p* ≤ 0.05 and 0.05 < *p* ≤ 0.10, respectively.

## 3. Results

### 3.1. White Blood Cell Profile and Serum Inflammatory Mediators

ETEC infection increased (*p* < 0.05) the white blood cell (WBC), neutrophil, lymphocyte, and monocyte counts on d 2, d 5, and d 14 PI when compared with d 0 pre-inoculation. They reached the peak on d 5 PI, followed by a reduction (*p* < 0.05) on d 14 PI (Table 1). On d 0 (pre-inoculation), there were no significant differences in total WBC count among treatments. However, pigs fed carbadox exhibited the highest (*p* < 0.05) lymphocyte and basophil counts, while pigs receiving 2% Asp showed the highest (*p* < 0.05) eosinophil counts among all groups on d 0 (Table 2). Pigs fed carbadox tended to have the lowest (*p* = 0.098) serum CRP concentration among all groups and had lower (*p* = 0.098) levels than pigs fed with NC, PC, or 2% Asp on d 0.

On d 2 PI, pigs fed with 2% Glu tended to have lower (*p* = 0.096) lymphocyte counts compared with pigs fed 2% Asp and carbadox, and tended to have the lowest (*p* = 0.096) lymphocyte counts among all groups. Additionally, the neutrophil-to-lymphocyte (Neu:Lym) ratio was affected by dietary treatments. Pigs fed 1% Glu or carbadox had the lowest (*p* < 0.05), while pigs fed 2% Asp had the highest (*p* < 0.05) Neu:Lym ratio on d 2 PI, compared with the other groups.

On d 5 PI, pigs fed 2% Glu tended to have higher (*p* = 0.067) neutrophil counts compared with pigs fed NC, 1% Glu, 1% Asp, and carbadox, and tended to have the highest (*p* = 0.067) neutrophil counts among all treatments. Pigs fed with 1% Glu showed similar trends in Neu:Lym ratio, neutrophil percentage, and lymphocyte percentage as pigs in NC and carbadox groups on d 5 PI (Supplementary Table S4). Pigs fed 2% Glu had the highest (*p* < 0.05) neutrophil percentage and lowest (*p* < 0.05) lymphocyte percentage, while those fed carbadox had the lowest (*p* < 0.05) neutrophil percentage and highest (*p* < 0.05) lymphocyte percentage among all groups. The Neu:Lym ratio followed a similar trend, being highest (*p* < 0.05) in the 2% Glu group and lowest (*p* < 0.05) in the carbadox group, compared with the other groups.

On d 14 PI, pigs fed 2% Glu tended to show the highest WBC (*p* = 0.064) and neutrophil (*p* = 0.090) counts among all groups, while pigs fed carbadox tended to show the lowest (*p* = 0.064 and *p* = 0.090) of both values. Pigs fed 1% Glu or 2% Asp showed WBC and neutrophil counts comparable to the NC and carbadox groups. Supplemented with 1% Asp or 2% Asp also tended to have higher (*p* = 0.059) eosinophil count compared with pigs in PC and carbadox groups on d 14 PI. Additionally, pigs in NC group exhibited the lowest (*p* < 0.05) basophil percentage among all groups (Supplementary Table S2).

Interestingly, when comparing 2% Glu supplementation with the carbadox group during peak infection, d 2 to d 5 PI, pigs fed 2% Glu consistently exhibited a lower (*p* < 0.05) lymphocyte percentage and a higher (*p* < 0.05) Neu:Lym ratio. From d 5 to d 14 PI, 2% Glu group also tended to show a consistent trend toward increased (*p* = 0.067 to 0.090) neutrophil counts and tended to have higher (*p* = 0.064) WBC on d 14 PI compared with the carbadox group. No differences in serum TNF-α, CRP, and haptoglobin levels were observed on d 2, 5, and 14 PI.

### 3.2. Red Blood Cell Profile

On d 0, 2% Asp supplementation showed the highest (*p* < 0.05) hemoglobin (HGB), hematocrit (HCT), mean corpuscular volume (MCV), mean corpuscular hemoglobin (MCH), and mean platelet volume (MPV), while pigs fed with 1% Glu had the lowest (*p* < 0.05) HGB, HCT, MCV, and total protein among all groups (Table 3).

On d 2 PI, pigs in the NC group exhibited the highest (*p* < 0.05) MCV, MCH, and mean corpuscular hemoglobin concentration (MCHC), while pigs in the carbadox group had the lowest (*p* < 0.05) MCV and MCH among all groups. In addition, pigs supplemented with 1% Asp showed the highest (*p* < 0.05) total protein, while pigs fed with 1% Glu had the lowest (*p* < 0.05) total protein among all groups on d 2 PI.

On d 5 PI, pigs fed with NC had the highest (*p* < 0.05), while pigs fed with carbadox had the lowest (*p* < 0.05) MCV among all groups. Additionally, pigs fed with NC tended to have the lowest (*p* = 0.085), while pigs fed with 1% Asp tended to have the highest (*p* = 0.085) total protein on d 5 PI. On d 14 PI, no differences were observed between pigs fed with NC and 2% Asp, and both groups exhibited the highest in HGB (*p* = 0.090), MCH (*p* < 0.05), and MCHC (*p* = 0.052) among all groups. In addition, pigs fed carbadox showed the lowest HGB (*p* = 0.090), MCV (*p* < 0.05), MCH (*p* < 0.05), and MPV (*p* < 0.05) among all groups on d 14 PI.

Interestingly, when compared with carbadox, pigs fed 2% Asp consistently showed distinct red blood cell parameters. On d 0, 2% Asp group had higher (*p* < 0.05) HGB, MCV, MCH, and MPV, compared with the carbadox group. On d 2 PI, pigs fed 2% Asp had higher (*p* < 0.05) MCV, MCH, and MCHC, compared with the carbadox group. On d 5 PI, pigs fed 2% Asp had higher (*p* < 0.05) MCV, compared with pigs fed carbadox. Lastly, on d 14 PI, 2% Asp group tended to have higher (*p* = 0.090) HGB, and had higher (*p* < 0.05) MCV, MCH, and MPV, compared with the carbadox group.

### 3.3. Immune-Related Gene Expression in Jejunal and Ileal Mucosa on d 14 Post-Inoculation

**Figure 1:**
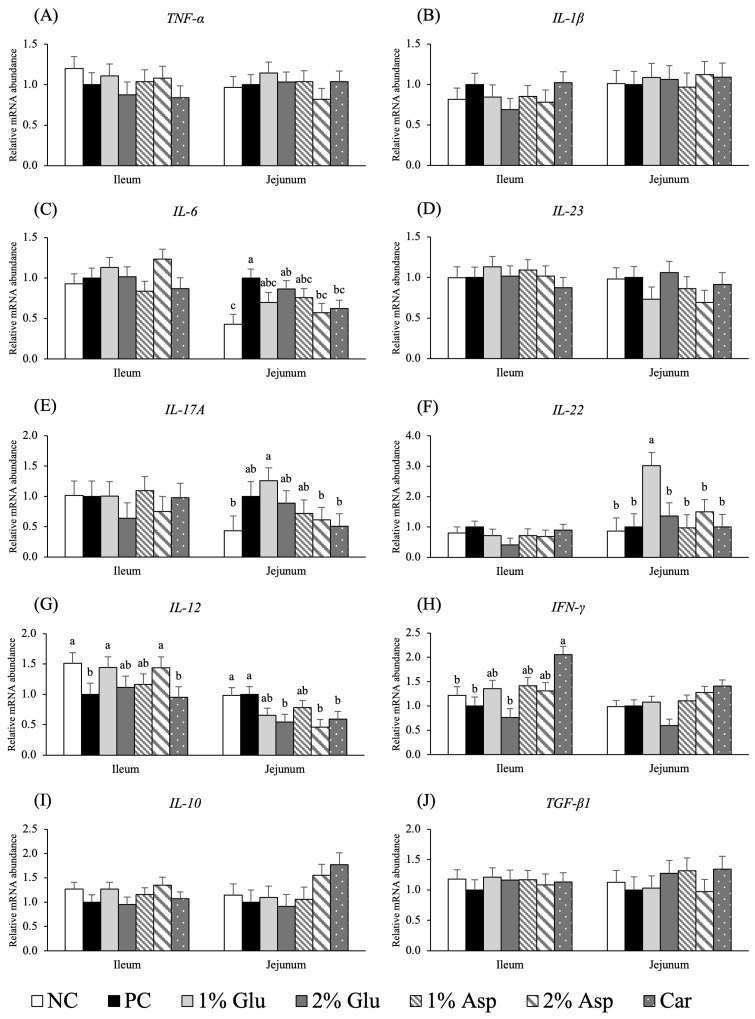
mRNA abundance of cytokines related to intestinal immune responses on d 14 post-inoculation. Relative mRNA abundance of *TNF-α* (**A**), *IL-1β* (**B**), *IL-6* (**C**), *IL-23* (**D**), *IL-17A* (**E**), *IL-22* (**F**), *IL-12* (**G**), *IFN-γ* (**H**), *IL-10* (**I**), *TGF-β1* (**J**) in jejunal and ileal mucosa of weaned pigs fed diets supplemented with glutamate (Glu), aspartate (Asp), or carbadox (Car) on d 14 PI. NC = negative control; PC = positive control; *TNF-α* = Tumor necrosis factor-alpha; *IL-1β* = Interleukin-1 beta; *IL-6* = Interleukin-6; *IL-23* = Interleukin-23; *IL-17A* = Interleukin-17A; *IL-22* = Interleukin-22; *IL-12* = Interleukin-12; *IFN-γ* = Interferon-gamma; *IL-10* = Interleukin-10; *TGF-β1* = Transforming growth factor-beta 1. Each least squares mean represents 7 replicates. ^a,b,c^ Means without a common superscript are different (*p* < 0.05).

Pigs fed 2% Asp or carbadox had lower (*p* < 0.05) *IL-6* mRNA abundance in the jejunal mucosa compared with pigs in the PC group (Figure 1C). Also, the *IL-6* mRNA abundance in pigs fed 1% Glu, 1% Asp, or 2% Asp did not differ from that observed in the NC or carbadox groups. No difference was observed in the mRNA abundance of *IL-17A* and *IL-22* in the ileal mucosa among all groups. Pigs supplemented with 1% Glu had higher (*p* < 0.05) mRNA abundance of *IL-17A* and *IL-22* in jejunal mucosa compared with pigs fed with NC, 2% Asp, and carbadox, and showed the highest (*p* < 0.05) mRNA abundance of both *IL-17A* and *IL-22* among all groups (Figure 1E,F). Pigs fed NC, 1% Glu, or 2% Asp had higher (*p* < 0.05) mRNA abundance of *IL-12* in ileal mucosa than pigs in the PC and carbadox groups, while pigs fed with 2% Glu, 2% Asp, or carbadox had lower (*p* < 0.05) mRNA abundance of *IL-12* in jejunal mucosa than pigs in the PC and NC groups (Figure 1G). In addition, compared with pigs receiving carbadox, pigs fed NC, PC, and 2% Glu showed lower (*p* < 0.05) mRNA abundance of *IFN-γ* in ileal mucosa (Figure 1H). However, no difference was observed in the mRNA abundance of *IFN-γ* in the jejunal mucosa among all groups. Additionally, no difference was observed in the mRNA abundance of *TNF-α*, *IL-1β*, *IL-10*, *TGF-β*, and *IL-23* in ileal and jejunal mucosa among all groups on d 14 PI.

### 3.4. Metabolomic Profiling of Serum on d 5 Post-Inoculation

A total of 345 metabolites were detected in serum samples on d 5 PI, including 136 identified and 209 unidentified metabolites. Based on the identified metabolites, a Partial Least Squares Discriminant Analysis (PLS-DA) score plot with 95% confidence intervals (represented by circled areas) revealed separation between the treatment groups (Figure S1). To further investigate the metabolic differences among the seven dietary treatments, pairwise comparisons were conducted. However, only a subset of metabolites showed significant differences between specific treatment groups, as illustrated in the PLS-DA score plots in Figure S2, particularly the NC group compared to 1% Glu group (Figure S2A), the NC group compared to 1% Asp group (Figure S2B), and the NC group compared to the carbadox group (Figure S2C). Compared with pigs in the NC group, pigs fed with 1% Glu, 1% Asp, or carbadox showed up-regulation (*p* < 0.05, FDR < 0.2; fold change > 2.0) of inositol-4-monophosphate and cholesterol in serum on d 5 PI. Additionally, pigs fed with 1% Glu exhibited increased levels (*p* < 0.05, FDR < 0.2; fold change > 2.0) of several other metabolites, including inosine, guanosine, tocopherol alpha-, conduritol-beta-epoxide, and pinitol compared with the NC group (Table 4).

**Figure 2:**
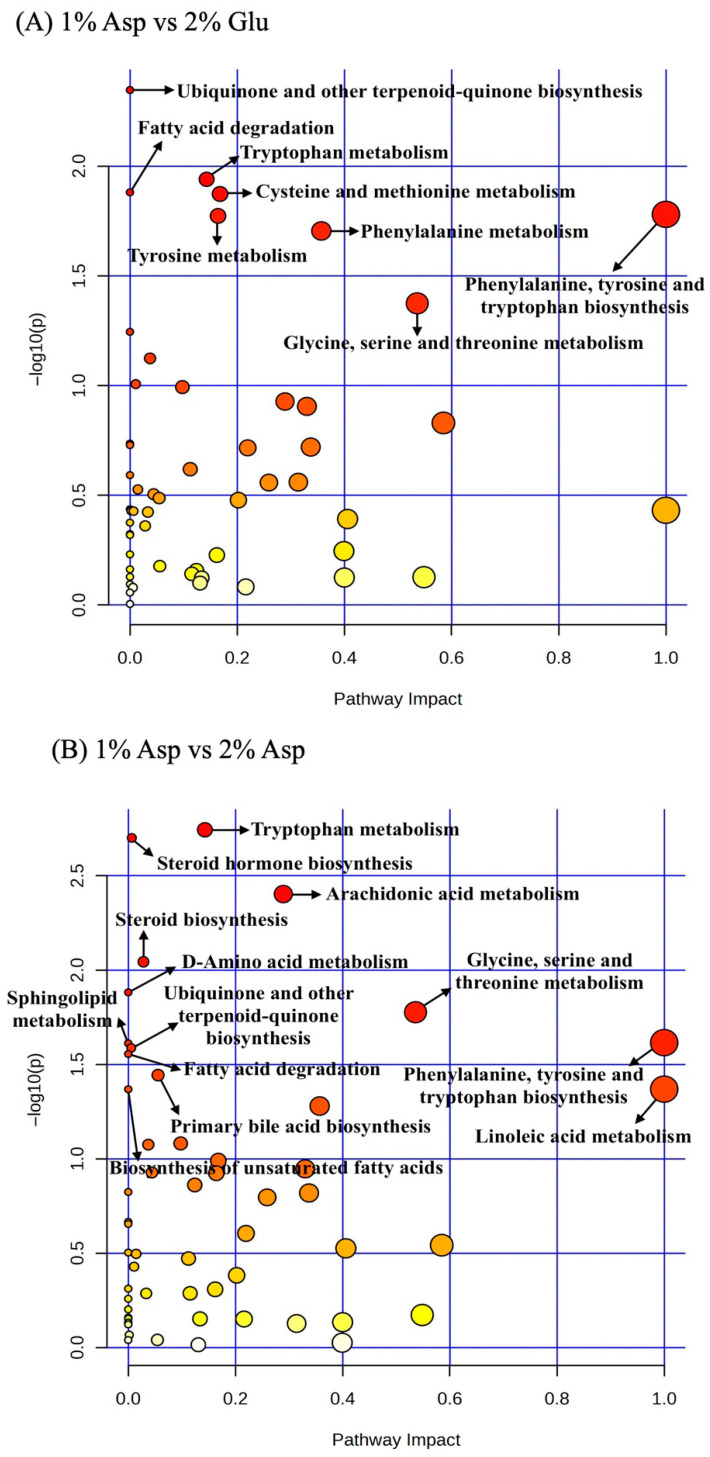
Pathway impact in serum samples on d 5 PI between treatment groups. Pathway impact in serum samples from d 5 PI between 1% Asp and 2% Glu groups (**A**), and 1% Asp and 2% Asp groups (**B**). Each treatment included 7 replicates. The x-axis represents the pathway impact values, and the y-axis represents the −log *p*-values from the pathway enrichment analysis. Dot color represents pathway significance based on −log10(*p*-value), with darker red indicating higher significance and lighter yellow indicating lower significance. Dot size reflects pathway impact. Arrows highlight selected pathways of biological relevance discussed in the text. Glu = glutamate; Asp = aspartate.

Pathway enrichment analysis revealed several significantly impacted pathways when comparing treatment groups (Figure 2). In the comparison of 1% Asp group to 2% Glu group, five pathways met the significance criteria (*p* < 0.05, FDR < 0.2, impact > 0.1), including phenylalanine, tyrosine and tryptophan biosynthesis (impact = 1.000), phenylalanine metabolism (impact = 0.357), cysteine and methionine metabolism (impact = 0.168), tyrosine metabolism (impact = 0.164), and tryptophan metabolism (impact = 0.143) (Table 5). Similarly, when comparing 1% Asp group with 2% Asp group, five enriched pathways were also identified, including phenylalanine, tyrosine and tryptophan biosynthesis (impact = 1.000), linoleic acid metabolism (impact = 1.000), glycine, serine and threonine metabolism (impact = 0.536), arachidonic acid metabolism (impact = 0.289), and tryptophan metabolism (impact = 0.143).

### 3.5. Metabolomic Profiling of Ileal Mucosa on d 14 Post-Inoculation

A total of 432 metabolites were detected in the ileal mucosa samples on d 14 PI, including 158 identified and 274 unidentified metabolites. The PLS-DA score plot with 95% confidence intervals showed separation among the seven groups, as illustrated in Figure S3. Several metabolites in ileal mucosa samples on d 14 PI differed significantly between treatment groups, as illustrated in the PLS-DA score plots in Figure S4, including the NC group compared to carbadox group (Figure S4A), the NC group compared to 1% Asp group (Figure S4B), and 2% Asp group compared to carbadox group (Figure S4C). Compared with the NC group, pigs fed with carbadox showed marked up-regulation (*p* < 0.05, FDR < 0.2; fold change > 2.0) of several metabolites, including homovanillic acid, conduritol-beta-epoxide, pinitol, 5-aminovaleric acid, cis-sinapinic acid, pentitol, inosine, glucose, ribitol, xylose, nicotinic acid, sinapinic acid, nicotianamine, perseitol, tyrosol, vanillic acid, ferulic acid, cis-p-coumaric acid, and indole-3-acetate (Table 4). Notably, homovanillic acid, 5-aminovaleric acid, and inosine were among the most up-regulated metabolites with the lowest FDR values. In contrast, levels of aminomalonic acid and glycine were reduced (*p* < 0.05, FDR < 0.2; fold change < 1.0) in the carbadox group. In the 1% Asp group, pinitol, cis-sinapinic acid, panose, maltotriose, perseitol, beta-alanine, and vanillic acid were significantly elevated (*p* < 0.05, FDR < 0.2; fold change > 2.0) compared with the NC group. Compared with the carbadox group, pigs supplemented with 2% Asp showed reduced (*p* < 0.05, FDR < 0.2; fold change < 1.0) levels of inosine and indole-3-acetate.

**Figure 3:**
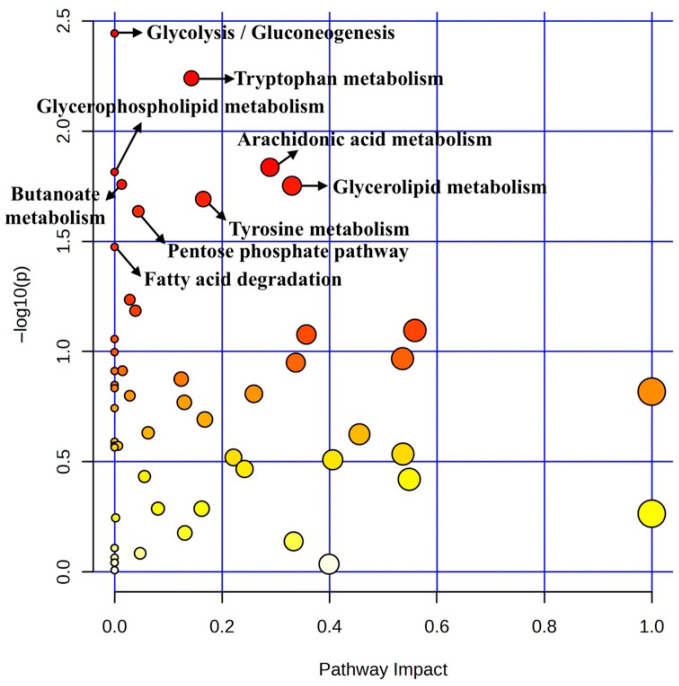
Pathway impact in ileal mucosa on d 14 PI between treatment groups. Pathway impact in ileal mucosa samples from d 14 PI between 1% Glu and carbadox groups. Each treatment included 7 replicates. The x-axis represents the pathway impact values, and the y-axis represents the −log *p*-values from the pathway enrichment analysis. Dot color represents pathway significance based on −log10(*p*-value), with darker red indicating higher significance and lighter yellow indicating lower significance. Dot size reflects pathway impact. Arrows highlight selected pathways of biological relevance discussed in the text. Glu = glutamate; Car = carbadox.

Pathway analysis of ileal mucosa on d 14 PI revealed that pigs fed with 1% Glu showed significant alterations compared with pigs fed with carbadox in multiple metabolic pathways (*p* < 0.05, FDR < 0.2, impact > 0.1), as reflected by the separation in the PLS-DA score plot in Figure 3, including glycerolipid metabolism, arachidonic acid metabolism, tyrosine metabolism, and tryptophan metabolism (Table 5).

### 3.6. Metabolomic Profiling of Ileal Digesta on d 14 Post-Inoculation

A total of 391 metabolites were detected in ileal digesta samples on d 14 PI, including 174 identified and 217 unidentified metabolites. The PLS-DA score plot with 95% confidence intervals showed separation among the seven groups, as illustrated in Figure S5. Untargeted metabolomics analysis of the ileal digesta revealed that pigs fed 1% Glu exhibited significant changes in metabolite profiles compared with the NC, PC, carbadox, and 2% Glu groups (*p* < 0.05, FDR < 0.2), as illustrated in the PLS-DA score plots in Figure S6. Compared with the PC group, pigs fed 1% Glu showed decreased (*p* < 0.05, FDR < 0.2; fold change < 1.0) levels of several metabolites, including cholesterol, palmitoleic acid, inositol-4-monophosphate, fructose-1-phosphate, oxalic acid, oleic acid, and arachidonic acid (Table 4). In comparison to the NC group, pigs fed 1% Glu had elevated (*p* < 0.05, FDR < 0.2; fold change > 2.0) concentrations of malonic acid, 5-aminovaleric acid, valine, and methionine, while levels of various metabolites were markedly reduced, including palmitoleic acid, threonic acid, lanosterol, 2,5-dihydroxypyrazine, cholesterol, arachidonic acid, methanolphosphate, inositol-4-monophosphate, 1-monopalmitin, zymosterol, icosenoic acid, oleic acid, pyrophosphate, oxalic acid, and fructose-1-phosphate. Compared with carbadox, supplemented with 1% Glu had decreased (*p* < 0.05, FDR < 0.2; fold change < 1.0) the levels of 2-hydroxybutanoic acid, N-acetylaspartic acid, inositol-4-monophosphate, oxalic acid, and taurine. Notably, cholesterol, palmitoleic acid, inositol-4-monophosphate, fructose-1-phosphate, oxalic acid, oleic acid, and arachidonic acid were consistently downregulated (*p* < 0.05, FDR < 0.2; fold change < 1.0) in pigs fed 1% Glu compared with both NC and PC groups. Similar reductions (*p* < 0.05, FDR < 0.2; fold change < 1.0) in cholesterol, methanolphosphate, inositol-4-monophosphate, pyrophosphate, and oxalic acid were also observed when comparing 1% Glu to 2% Glu.

**Figure 4:**
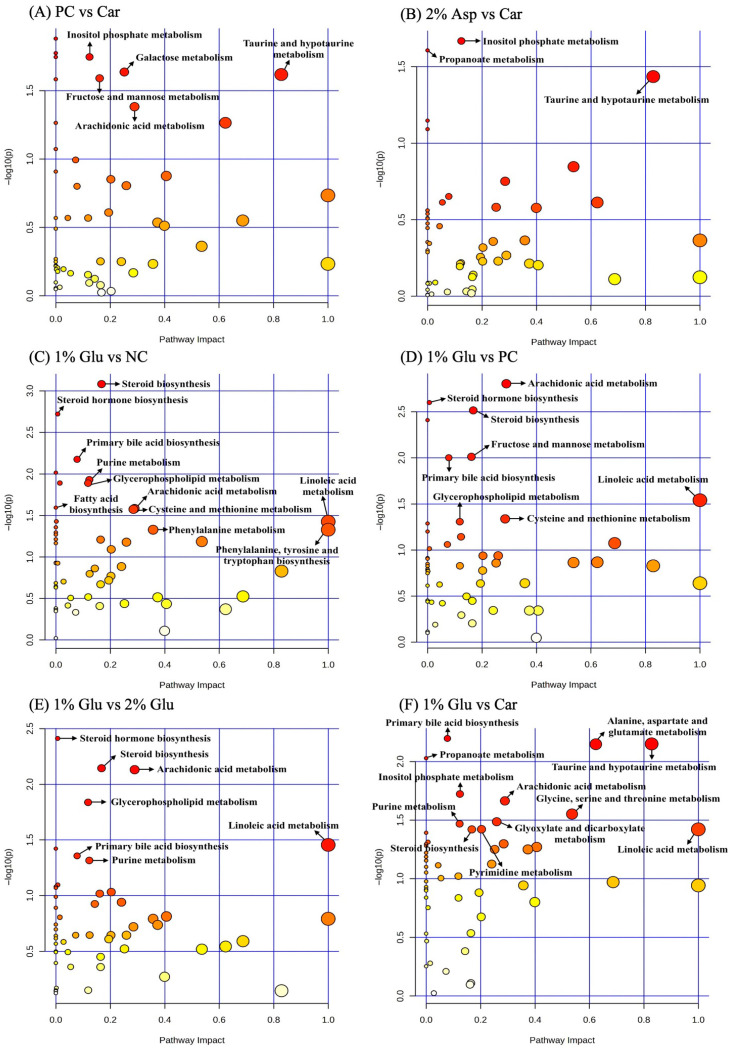
Pathway impact in ileal digesta samples on d 14 PI between treatment groups. Pathway impact in ileal digesta samples from d 14 PI between PC and Car groups (**A**), 2% Asp and Car groups (**B**), 1% Glu and NC groups (**C**), 1% Glu and PC groups (**D**), 1% Glu and 2% Glu groups (**E**), and 1% Glu and Car groups (**F**). Each treatment included 7 replicates. The x-axis represents the pathway impact values, and the y-axis represents the −log *p*-values from the pathway enrichment analysis. Dot color represents pathway significance based on −log10(*p*-value), with darker red indicating higher significance and lighter yellow indicating lower significance. Dot size reflects pathway impact. Arrows highlight selected pathways of biological relevance discussed in the text. NC = negative control; PC = positive control; Car = carbadox; Glu = glutamate; Asp = aspartate.

Pathway enrichment analysis of ileal digesta on d 14 PI revealed several significantly impacted pathways across treatment comparisons (*p* < 0.05, FDR < 0.2, impact > 0.1) (Figure 4). In the comparison of the PC group compared with the carbadox group, four pathways were significantly enriched, including taurine and hypotaurine metabolism (impact = 0.829), galactose metabolism (impact = 0.252), fructose and mannose metabolism (impact = 0.161), and inositol phosphate metabolism (impact = 0.124) (Table 5). When comparing the PC group to 1% Glu group, three pathways met significance criteria, including arachidonic acid metabolism (impact = 0.289), steroid biosynthesis (impact = 0.168), and fructose and mannose metabolism (impact = 0.161). In the comparison of the NC to 1% Glu group, significant enrichment was observed in linoleic acid metabolism (impact = 1.000), phenylalanine, tyrosine and tryptophan biosynthesis (impact = 1.000), phenylalanine metabolism (impact = 0.357), arachidonic acid metabolism (impact = 0.289), cysteine and methionine metabolism (impact = 0.285), steroid biosynthesis (impact = 0.168), purine metabolism (impact = 0.123), and glycerophospholipid metabolism (impact = 0.118). For the comparison of 2% Asp group to the carbadox group, four pathways were significantly impacted, including taurine and hypotaurine metabolism (impact = 0.829), alanine, aspartate and glutamate metabolism (impact = 0.623), arginine biosynthesis (impact = 0.406), and glyoxylate and dicarboxylate metabolism (impact = 0.259). Furthermore, the comparison of 1% Glu group to carbadox group revealed significant enrichment in linoleic acid metabolism (impact = 1.000), taurine and hypotaurine metabolism (impact = 0.829), alanine, aspartate and glutamate metabolism (impact = 0.623), glycine, serine and threonine metabolism (impact = 0.536), arachidonic acid metabolism (impact = 0.289), glyoxylate and dicarboxylate metabolism (impact = 0.259), pyrimidine metabolism (impact = 0.203), steroid biosynthesis (impact = 0.168), inositol phosphate metabolism (impact = 0.124), and purine metabolism (impact = 0.123). Lastly, when comparing 1% Glu group to 2% Glu group, arachidonic acid metabolism (impact = 0.289), steroid biosynthesis (impact = 0.168), and glycerophospholipid metabolism (impact = 0.118) were differentially enriched (Table 5).

## 4. Discussion

Weaning imposes physiological and psychological stress on piglets, disrupting gut function, impairing immunity, and reducing nutrient utilization, which compromises growth and increases susceptibility to pathogens like ETEC [3,19,26]. Glu and Asp are classified as non-essential amino acids under normal conditions, yet their roles in metabolism and cellular processes become increasingly important during stress [10,20]. Both serve as key energy sources for enterocytes and intermediates in amino acid metabolism [15,27,28]. In states of reduced feed intake and heightened physiological demand, they may serve as conditionally essential nutrients to support immune modulation, intestinal repair, and energy metabolism [14,16,17]. Our previously published research reported that supplementation with Glu or Asp has alleviated PWD in piglets challenged with F18 ETEC [21]. In this follow-up study, we observed that these amino acids contributed to the modulation of both systemic and mucosal immune responses. Furthermore, metabolic profiling revealed distinct alterations in pathways associated with amino acid interconversion, energy metabolism, and inflammation, indicating that Glu and Asp played multifaceted roles in restoring immune and metabolic homeostasis under ETEC-induced diarrhea. These results extended previous evidence and highlighted the potential of targeted amino acid supplementation as a strategy to enhance resilience in weaned pigs, consistent with previous reports [12,18,29].

Infection with F18 ETEC induces systemic inflammation, resulting in elevated WBC and alterations in circulatory blood cell profiles in infected pigs [5]. In this study, 1% Glu supplementation reduced the Neu:Lym ratio in infected pigs on d 2 PI, similar to the response observed in the carbadox group. This suggested that Glu may help mitigate systemic inflammation and support immune homeostasis comparable to the effects of an antibiotic, consistent with improved growth performance in our previous study [21]. However, supplementation with 2% Glu decreased lymphocyte counts post-inoculation, alongside increased WBC and neutrophil counts, as well as an elevated Neu:Lym ratio following infection, aligning with reduced productivity in the previous report [21]. This indicated excessive Glu may impair microbial clearance by suppressing T lymphocyte proliferation, essential for combating ETEC [30,31]. In contrast, supplementation with Asp increased the Neu:Lym ratio compared to the carbadox group on d 2 and d 5 PI, indicating a heightened inflammatory response in infected pigs, followed by reduced WBC and neutrophil counts on d 14 PI, paralleling the carbadox group. Given that Asp also alleviates diarrhea and improves growth [21,32,33], it may facilitate early clearance of bacterial infection by promoting immune cell proliferation and activation. These findings suggested distinct roles of Glu and Asp, warranting further investigation into their mechanisms.

Hematological indices provide valuable insights into systemic responses following ETEC infection, as diarrhea can lead to alterations in red blood cell parameters [34,35]. In this study, bacterial challenge affected MCV, MCH, MCHC, and total protein. On d 2 PI, these indices were lower in carbadox and amino acid-supplemented groups than in NC pigs, indicating early changes in erythrocyte morphology and hemoglobin content. During peak diarrhea on d 5 PI, MCV remained reduced in 1% or 2% Glu, 1% Asp, and carbadox groups, suggesting sustained erythropoietic stress and further supporting possible inflammation-associated microcytic changes due to inadequate nutritional intake caused by diarrhea [36]. Total protein increased in the 1% Asp group, which may reflect physiological responses associated with diarrhea or altered protein metabolism [32,33,37]. Further investigation is needed to clarify this relationship. On d 14 PI, HGB, MCV, and MCH remained altered, with the lowest values in the carbadox group and partial normalization in the amino acid-supplemented pigs, especially with 2% Asp supplementation. Notably, 2% Asp increased MCV relative to the carbadox group, possibly by supporting nucleotide synthesis and erythropoiesis, leading to more reticulocytes [14,15,17]. These results indicated that Glu and Asp may potentially help stabilize red blood cell indices during recovery from ETEC challenge.

ETEC, a Gram-negative bacterium, colonizes the intestinal epithelium via fimbriae, and its enterotoxins induce secretory diarrhea [4]. Its lipopolysaccharide (LPS) activates Toll-like receptor 4 (TLR4) and nuclear factor kappa-light-chain-enhancer of activated B cells (NF-κB) signaling, elevating pro-inflammatory cytokines and driving T helper type 1 cell (Th1) and T helper type 17 cell (Th17) responses [38,39]. In this study, Glu or Asp supplementation modulated *IL-6* expression in jejunal mucosa, resembling NC and carbadox groups except with 2% Glu, suggesting anti-inflammatory potential comparable to antibiotics. This aligned with reports that Glu and Asp suppressed TLR4, nucleotide-binding oligomerization domain (NOD), and NF-κB pathways in LPS-challenged pigs [17,29]. Glu and Asp supplementation also influenced Th1 cytokines (*IL-12* and *IFN-γ*), which aid macrophage-mediated clearance of ETEC [33]. Notably, 2% Glu reduced *IFN-γ* expression in the ileum when compared with the carbodox group, consistent with evidence that high extracellular Glu impairs Th1 responses [30,31,40,41]. The modulation of *IL-6* and *IFN-γ* expression observed with Glu or Asp supplementation paralleled improved growth performance and lower diarrhea severity [21], suggesting transcriptional modulation may have contributed to these physiological outcomes.

Glu and Asp differentially regulated Th17 cytokines. 1% Glu supplementation increased *IL-17A* and *IL-22* expression in the jejunal mucosa, while Asp reduced them. Since Th17 cytokines are typically upregulated during ETEC infection [39], the reduced Th17 cytokine expression with Asp supplementation aligned with the lower diarrhea incidence reported previously [21], indicating Asp may aid early bacterial clearance by stimulating immune cell proliferation through nucleotide synthesis [17]. Conversely, 1% Glu sustained Th17 expression through d 14 PI, paralleling improved growth and reduced bacterial shedding observed in the previous report [21], indicating modulation rather than suppression of inflammation. These results supported previous evidence that Glu promoted gut health and immune regulation [16,28,30]. Collectively, Glu and Asp may protect against ETEC and reduce gut injury through distinct mechanisms. Together with the hematological and metabolomic data, these mRNA abundance patterns suggested that Glu or Asp supplementation may alleviate ETEC-induced inflammation and restore immune–metabolic homeostasis, especially as Glu supplementation was associated with reduced neutrophil counts following infection. As only mRNA abundance was assessed, these findings should be interpreted cautiously. Protein-level or functional assays are needed to confirm immune effects, and the use of a single housekeeping gene in RT-qPCR limits data reliability; therefore, employing multiple reference genes is recommended.

ETEC infection disrupts host metabolism, leading to systemic and enteric metabolic alterations, while dietary supplementation further modulates these metabolic responses, as reflected in distinct serum and ileal metabolite profiles [6,42]. The present study provided a comprehensive metabolomic profiling of serum, ileal mucosa, and ileal digesta in F18 ETEC-challenged weaned pigs fed Glu, Asp, or carbadox. Notably, supplementation with 1% Glu or 1% Asp increased serum levels of inositol-4-monophosphate and cholesterol during peak diarrhea on d 5 PI. Inositol phosphates are key regulators of intracellular signaling, energy balance, membrane dynamics, and osmoregulation [43,44], and their increased abundance may contribute to improved tolerance to diarrhea severity and enhanced expression of intestinal barrier-related genes [21]. The elevation of cholesterol may reflect membrane remodeling in enterocytes or immune cells, shifts in lipid metabolism, or changes in steroid hormone production during infection [45], which may be linked to the intestinal structural changes reported previously [21]. These changes may indicate that Glu and Asp supplementation may enhance cellular responses to bacterial challenge and support gut barrier repair, contributing to improved host resilience. Supplementation with 1% Glu increased levels of inosine and guanosine, which are purine metabolites linked to energy metabolism and immune-related processes [46]. Elevated α-tocopherol (vitamin E) in 1% Glu-fed pigs could suggest enhanced antioxidant defenses, potentially mitigating ETEC-induced oxidative stress [18,47]. These metabolic changes highlighted the potential protective and restorative effects of Glu on systemic metabolism, suggesting improved nucleotide turnover and immune functions under stress conditions, which may contribute to improved growth performance [21].

Pathway analysis of serum metabolites on d 5 PI suggested enrichment of several amino acid metabolism pathways when comparing 2% Glu and 2% Asp supplementation with 1% Asp. These pathways included phenylalanine, tyrosine, tryptophan, glycine, serine, threonine, cysteine, and methionine metabolism. These amino acids are involved in protein synthesis and antioxidant production and may contribute to mucosal repair and immune responses during infection-induced oxidative stress [48,49]. Pathway analysis also suggested enrichment of linoleic acid and arachidonic acid metabolism in the comparison between 1% Asp and 2% Asp groups, which may indicate potential involvement of lipid mediator pathways associated with inflammatory responses [50]. These pathway-level observations were consistent with the modulation of immune responses and reduced diarrhea severity observed following Glu or Asp supplementation [21]. However, interpretation of pathway enrichment should be made cautiously because only a limited number of metabolites were mapped to some pathways.

Metabolomic profiling of the ileal mucosa on d 14 PI, during the recovery period, revealed notable metabolic alterations associated with both infection and dietary interventions. Several detected metabolites showed significant changes between treatment groups, suggesting that dietary supplementation continued to shape intestinal metabolism well beyond the acute phase of infection. Carbadox supplementation led to a pronounced upregulation of several metabolites, including phenolic compounds with known antioxidant capacity (e.g., homovanillic acid, ferulic acid, vanillic acid, tyrosol), polyols (e.g., ribitol, xylose, pinitol), nucleoside derivatives (inosine), and microbial-related metabolites (conduritol-beta-epoxide, 5-aminovaleric acid, indole-3-acetate) compared with the NC group [6,51,52]. These changes suggested enhanced antioxidant activity, microbial fermentation, and possibly altered host–microbe interactions of carbadox [53]. Interestingly, carbadox-treated pigs showed reduced levels of aminomalonic acid, a biomarker of oxidative stress, and glycine, a precursor for glutathione, compared with the NC group [10,14,54]. This may indicate that these pigs experienced lower levels of mucosal oxidative stress post-ETEC challenge, despite the NC group not being challenged. The antimicrobial action of carbadox likely reduced pathogen load and secondary inflammation, contributing to improved diarrhea outcomes observed in the previous report [21]. These changes highlighted a complex interplay between antimicrobial treatment, microbial composition, and host metabolism.

Supplementation with 1% Asp also upregulated carbohydrate-related metabolites, including maltotriose and panose, likely derived from host metabolism, and pinitol and perseitol, typically originating from plant-based diets, compared with the NC group, suggesting alterations in carbohydrate metabolism in ileal mucosa on d 14 PI [55,56,57]. These changes may reflect enhanced enzymatic digestion, improved mucosal energy supply, increased fermentation by beneficial microbes, or more efficient mucosal carbohydrate handling. Further studies on nutrient digestibility, the expression of carbohydrate-digesting enzymes, and carbohydrate-fermenting microbes are recommended to clarify these effects. Beta-alanine, cis-sinapinic acid, and vanillic acid possess antioxidant and anti-inflammatory properties [58,59]. Their upregulation suggested that Asp supplementation may help mitigate oxidative stress during infection, aligning with our previous findings that Asp reduced the frequency of severe diarrhea in ETEC-infected piglets [21]. Notably, 2% Asp supplementation reduced inosine and indole-3-acetate levels compared to carbadox, suggesting a divergence from antibiotic-associated effects, particularly in nucleotide and microbial indole metabolism.

Pathway analysis of ileal mucosa metabolites on d 14 PI suggested enrichment of several metabolic pathways in pigs supplemented with 1% Glu compared with carbadox treatment, including glycerolipid and arachidonic acid metabolism. Glycerolipid metabolism is involved in lipid turnover and cellular energy production [60]. A reduction in glyceric acid was observed in pigs fed 1% Glu compared with the carbadox group (*p* < 0.01, FDR = 0.263), which may indicate altered energy metabolism. Glu is known to serve as an energy substrate for enterocytes and contribute carbon skeletons to the TCA cycle during intestinal stress [11]. Arachidonic acid metabolism is associated with inflammatory responses and immune regulation [61], and a decrease in arachidonic acid levels was observed in pigs fed 1% Glu compared with the carbadox group (*p* < 0.05, FDR = 0.264). This trend may suggest potential modulation of inflammatory pathways during infection [16,30,31]. Consistent with this observation, Glu supplementation was associated with altered cytokine responses, reduced bacterial shedding and improved growth performance [21]. Lower levels of indole-3-acetate (a tryptophan metabolite) and homovanillic acid (a tyrosine metabolite) were also observed in pigs supplemented with 1% Glu compared with the carbadox group (*p* < 0.01, FDR = 0.264). These changes may reflect alterations in microbial metabolism or host stress responses [62,63,64]. However, interpretation of these pathway-level findings should be made cautiously because several comparisons exhibited relatively high FDR values.

Future studies with larger sample sizes and integrative omics approaches will be necessary to confirm these trends and establish the mechanistic basis of the potential effects of Glu. Together, these results suggested that both amino acid supplementation and antibiotic treatment led to distinct and sustained changes in the ileal mucosal metabolome following ETEC infection. While carbadox induced widespread shifts likely tied to antimicrobial effects and host-microbiota interactions, Glu and Asp supplementation elicited more targeted alterations in host cells, possibly contributing to mucosal recovery and metabolic homeostasis through different mechanisms.

Untargeted metabolomics of ileal digesta on d 14 PI revealed marked shifts with 1% Glu supplementation. Pigs fed 1% Glu showed distinct metabolic changes compared with PC, NC, carbadox, and 2% Glu groups, with multiple differentially abundant metabolites. Several lipids, energy intermediates, and amino acid derivatives were significantly modulated. Notably, cholesterol, palmitoleic acid, inositol-4-monophosphate, fructose-1-phosphate, oxalic acid, oleic acid, and arachidonic acid were consistently downregulated versus the PC and NC groups. These metabolites are linked to lipid metabolism, energy balance, and inflammation [43,65,66]. For example, reduced arachidonic acid, a leukotriene precursor, suggested an anti-inflammatory potential of 1% Glu supplementation [67,68], which is consistent with the reduced neutrophil counts observed in this study. Similarly, decreased levels of cholesterol and steroid precursors, including lanosterol and zymosterol, may indicate alterations in sterol biosynthesis in response to dietary intervention. Cholesterol plays a key role in membrane remodeling and cell proliferation [69]. The observed reduction in cholesterol and its precursors may suggest a slower rate of epithelial cell turnover in the ileum, potentially due to reduced tissue damage and injury from infection, consistent with the ileal changes reported previously in piglets supplemented with 1% Glu [21]. Since these metabolites can be derived from both the host and microbes [70,71], interpretation requires integration with microbiota data.

In contrast, 1% Glu increased amino acid-related metabolites, including valine, methionine, and 5-aminovaleric acid, compared with the NC group. Their accumulation may reflect reduced absorption, altered microbial metabolism, or enhanced luminal availability. As valine and methionine are essential for protein synthesis, their presence in digesta suggests incomplete utilization by the host [72,73]. This pointed to a shift in nitrogen metabolism in the gut lumen post-infection. Further studies are needed to determine whether these changes translated into improved mucosal repair or protein synthesis under ETEC challenge, such as systemic amino acid profiling or intestinal transcriptomics of amino acid transporters [74].

Additionally, 1% Glu lowered inositol-4-monophosphate and oxalic acid relative to all other groups. Reduced levels of inositol-4-monophosphate may reflect decreased immune cell signaling activities, potentially indicating reduced inflammation or tissue damage [75], consistent with the reduced neutrophil counts and modulation of cytokine responses observed in this study. Similarly, lower oxalic acid concentrations may suggest reduced oxidative stress and improved microbial balance [76,77]. Together, these changes pointed to potential roles of Glu supplementation in enhancing intestinal integrity and promoting a healthier gut environment under infection conditions [16], as evidenced by reduced bacterial shedding, improved expression of intestinal integrity–related genes, and regulated intestinal cytokine responses with 1% Glu supplementation [21].

Pathway analysis of ileal digesta metabolites on d 14 PI suggested enrichment of several lipid and amino acid metabolism pathways in comparisons involving 1% Glu supplementation. These included arachidonic acid metabolism and steroid biosynthesis, which are associated with inflammatory lipid mediators and sterol metabolism. Comparisons with the NC group also suggested enrichment of phenylalanine, tyrosine, and tryptophan biosynthesis pathways, which provide precursors for protein synthesis and neurotransmitters such as dopamine, norepinephrine, epinephrine, and serotonin [50,78]. Glu has been reported to function as a signaling molecule in the enteric nervous system and may influence neuroendocrine responses in the gastrointestinal tract [79]. Pathway analysis also suggested enrichment of methionine and cysteine metabolism, and increased methionine levels were observed. Methionine, cysteine, and Glu contribute to glutathione synthesis and are associated with antioxidant pathways and intestinal barrier function [21,80]. Comparisons between Glu or Asp supplementation and carbadox group further suggested enrichment of pathways related to alanine, aspartate, and glutamate metabolism, taurine and hypotaurine metabolism, and glyoxylate and dicarboxylate metabolism. These pathways reflected the metabolic roles of Glu and Asp in amino acid interconversion and energy metabolism, including contributions to the TCA cycle that may support enterocyte energy production, creating a distinct metabolic profile compared to antibiotic treatment [11,12,81]. Comparison between 1% and 2% Glu suggested that higher Glu supplementation did not result in additional metabolic shifts under the conditions of this study, suggesting that 1% Glu might be a more optimal dose under the experimental conditions. Because some pathways were supported by a limited number of mapped metabolites, these pathway-level observations should be interpreted cautiously.

## 5. Conclusions

This study highlighted the important roles of Glu and Asp as functional amino acids that may become conditionally essential during stress periods, particularly weaning, in piglets infected with ETEC. Supplementation with Glu or Asp revealed immunomodulatory and metabolic benefits, including the attenuation of systemic and intestinal inflammation, stabilization of hematological parameters, and potential support of immune cell functions. Notably, 1% Glu supplementation showed effects comparable to carbadox in reducing inflammation, while 1–2% Asp appeared to aid recovery through pathways that may be distinct from those of Glu. These findings suggested that targeted nutritional strategies involving Glu and Asp could help support gut health and resilience, providing a promising approach to reduce antibiotic use in swine production and contribute to more sustainable animal husbandry practices.

## Figures and Tables

**Table 1:** Total and differential white blood cell and red blood cell profiles of ETEC-challenged weaned piglets fed experimental diets.

Item ^1^	d 0	d 2 PI	d 5 PI	d 14 PI	SEM	*p*-Value
WBC, 10^3^/μL	8.68 ^c^	9.98 ^b^	12.37 ^a^	9.77 ^b^	0.464	<0.001
Neu, 10^3^/μL	4.04 ^c^	4.96 ^b^	6.27 ^a^	5.15 ^b^	0.403	<0.001
Lym, 10^3^/μL	3.68 ^b^	3.93 ^b^	4.53 ^a^	4.03 ^b^	0.191	<0.001
Mono, 10^3^/μL	0.63 ^b^	0.80 ^a^	0.88 ^a^	0.60 ^b^	0.063	<0.001
Eos, 10^3^/μL	0.25 ^a^	0.29 ^a^	0.22 ^a^	0.08 ^b^	0.033	<0.001
Baso, 10^3^/μL	0.01 ^b^	0.02 ^ab^	0.02 ^a^	0.02 ^ab^	0.002	0.028
Neu:Lym	1.07 ^c^	1.19 ^bc^	1.39 ^a^	1.29 ^ab^	0.066	0.002
RBC, 10^6^/μL	7.72 ^b^	8.05 ^a^	7.99 ^ab^	6.35 ^c^	0.130	<0.001
HGB, g/dL	8.23 ^a^	8.57 ^a^	8.36 ^a^	7.51 ^b^	0.151	<0.001
HCT, %	32.77 ^ab^	33.32 ^a^	31.50 ^b^	26.35 ^c^	0.507	<0.001
MCV, fL	42.51 ^a^	41.34 ^b^	39.67 ^c^	41.30 ^b^	0.568	<0.001
MCH, pg	10.63 ^b^	10.74 ^b^	10.44 ^b^	11.82 ^a^	0.189	<0.001
MCHC, g/dL	25.01 ^c^	25.98 ^b^	26.25 ^b^	28.41 ^a^	0.253	<0.001
RDW, %	29.46 ^a^	27.45 ^b^	26.53 ^c^	25.91 ^c^	0.686	<0.001
Platelets, 10^3^/μL	422.24 ^a^	390.59 ^a^	391.75 ^a^	319.16 ^b^	36.413	0.001
MPV, fL	7.82	7.74	7.90	7.62	0.205	0.3811
Total protein, g/dL	5.70 ^a^	5.87 ^a^	5.75 ^a^	4.96 ^b^	0.121	<0.001

^1^ WBC = white blood cell; Neu = neutrophil; Lym = lymphocyte; Mono = monocyte; Eos = eosinophil; Baso = basophil; Neu:Lym = neutrophil-to-lymphocyte ratio; RBC = red blood cell; HGB = hemoglobin; HCT = hematocrit; MCV = mean corpuscular volume; MCH = mean corpuscular hemoglobin; MCHC = mean corpuscular hemoglobin concentration; RDW = red cell distribution width; MPV = mean platelet volume; PI = post-inoculation. Each least squares mean represents 49 replicates. ^a,b,c^ Means without a common superscript are different (*p* < 0.05).

**Table 2:** White blood cell profile and serum inflammatory biomarkers of ETEC-challenged weaned piglets fed experimental diets.

Item ^1^	NC	PC	1% Glu	2% Glu	1% Asp	2% Asp	Carbadox	SEM	*p*-Value
**d** ** ** **0**									
WBC, 10^3^/μL	8.83	9.31	8.74	8.10	7.46	9.19	9.10	0.857	0.699
Neu, 10^3^/μL	3.97	4.74	3.75	3.60	3.66	4.61	3.84	0.637	0.763
Lym, 10^3^/μL	3.75 ^bc^	3.63 ^bcd^	4.12 ^ab^	3.37 ^cd^	3.10 ^d^	3.34 ^cd^	4.40 ^a^	0.257	0.003
Mono, 10^3^/μL	0.62	0.61	0.67	0.62	0.52	0.73	0.63	0.102	0.789
Eos, 10^3^/μL	0.29 ^ab^	0.32 ^ab^	0.12 ^b^	0.13 ^b^	0.18 ^b^	0.49 ^a^	0.19 ^b^	0.077	0.012
Baso, 10^3^/μL	0.01 ^b^	0.01 ^b^	0.01 ^b^	0.01 ^b^	0.01 ^b^	0.01 ^b^	0.04 ^a^	0.007	0.020
Neu:Lym	1.05	1.39	0.89	0.99	0.97	1.34	0.87	0.159	0.113
TNF-α, pg/mL	77.40	97.27	25.66	33.10	88.64	93.93	18.99	30.429	0.261
CRP, μg /mL	6.76 ^a^	6.01 ^a^	4.80 ^ab^	4.38 ^ab^	7.48 ^a^	4.64 ^ab^	2.34 ^b^	1.207	0.098
Hp, mg/mL	2.43	2.74	2.02	2.06	2.49	2.29	1.56	0.311	0.105
**d** ** ** **2** ** ** **PI**									
WBC, 10^3^/μL	9.78	8.78	9.91	10.19	9.58	11.11	10.44	1.141	0.809
Neu, 10^3^/μL	4.63	3.99	5.14	5.84	4.62	5.77	4.62	0.817	0.572
Lym, 10^3^/μL	4.06 ^ab^	3.77 ^ab^	4.05 ^ab^	3.07 ^b^	3.72 ^ab^	4.08 ^a^	4.70 ^a^	0.369	0.096
Mono, 10^3^/μL	0.72	0.75	0.73	0.72	0.88	0.93	0.91	0.153	0.822
Eos, 10^3^/μL	0.36	0.14	0.19	0.53	0.32	0.31	0.19	0.124	0.341
Baso, 10^3^/μL	0.01	0.02	0.01	0.02	0.02	0.02	0.01	0.005	0.261
Neu:Lym	1.17 ^abc^	1.04 ^bc^	0.95 ^c^	1.40 ^ab^	1.24 ^abc^	1.53 ^a^	0.99 ^c^	0.141	0.041
TNF-α, pg/mL	94.43	36.62	24.49	54.42	106.01	46.33	25.70	27.456	0.188
CRP, μg /mL	5.82	4.99	3.06	3.19	4.29	3.84	3.28	1.008	0.378
Hp, mg/mL	3.51	3.65	2.41	3.20	3.21	2.55	2.56	0.524	0.432
**d** ** ** **5** ** ** **PI**									
WBC, 10^3^/μL	11.99	12.78	12.89	13.95	10.66	12.99	11.49	0.972	0.268
Neu, 10^3^/μL	5.78 ^b^	6.88 ^ab^	5.69 ^b^	8.13 ^a^	5.46 ^b^	6.44 ^ab^	5.46 ^b^	0.747	0.067
Lym, 10^3^/μL	4.90	4.73	4.50	4.38	4.25	4.20	4.74	0.401	0.835
Mono, 10^3^/μL	1.03 ^a^	0.90 ^ab^	1.02 ^a^	0.62 ^b^	0.64 ^b^	1.00 ^a^	0.95 ^ab^	0.114	0.058
Eos, 10^3^/μL	0.27	0.25	0.28	0.34	0.18	0.15	0.08	0.078	0.280
Baso, 10^3^/μL	0.02	0.02	0.02	0.03	0.02	0.03	0.02	0.007	0.511
Neu:Lym	1.21 ^bc^	1.49 ^ab^	1.28 ^bc^	1.76 ^a^	1.36 ^abc^	1.55 ^ab^	1.09 ^c^	0.147	0.023
TNF-α, pg/mL	96.30	82.01	28.36	128.73	91.39	66.21	47.03	37.853	0.584
CRP, μg /mL	3.10	2.36	3.36	7.47	2.07	4.24	2.06	1.539	0.121
Hp, mg/mL	2.55	2.89	2.25	2.78	2.37	1.98	1.42	0.512	0.376
**d** ** ** **14** ** ** **PI**									
WBC, 10^3^/μL	9.59 ^b^	10.09 ^ab^	9.70 ^ab^	12.29 ^a^	10.40 ^ab^	8.73 ^b^	7.91 ^b^	1.218	0.064
Neu, 10^3^/μL	5.05 ^abc^	5.46 ^abc^	4.63 ^bc^	7.00 ^a^	5.90 ^ab^	4.38 ^bc^	3.66 ^c^	0.868	0.090
Lym, 10^3^/μL	3.80	3.95	4.43	4.75	4.08	3.69	3.61	0.463	0.283
Mono, 10^3^/μL	0.64	0.54	0.49	0.77	0.52	0.68	0.57	0.103	0.499
Eos, 10^3^/μL	0.08 ^ab^	0.05 ^b^	0.07 ^ab^	0.08 ^ab^	0.15 ^a^	0.13 ^a^	0.04 ^b^	0.029	0.059
Baso, 10^3^/μL	0.01	0.02	0.03	0.02	0.02	0.02	0.01	0.004	0.109
Neu:Lym	1.42	1.37	1.12	1.44	1.22	1.45	0.97	0.195	0.485
TNF-α, pg/mL	90.39	88.62	4.30	100.57	81.66	84.48	8.95	39.731	0.437
CRP, μg /mL	5.11	4.82	4.70	4.51	5.36	5.50	2.91	0.967	0.484
Hp, mg/mL	1.40	1.05	1.12	1.38	1.21	1.31	0.68	0.263	0.485

^1^ WBC = white blood cell; Neu = neutrophil; Lym = lymphocyte; Mono = monocyte; Eos = eosinophil; Baso = basophil; Neu:Lym = neutro-phil-to-lymphocyte ratio; TNF-α = tumor necrosis factor-alpha; CRP = C-reactive protein; Hp = haptoglobin; PI = post-inoculation. Each least squares mean represents 7 replicates. ^a,b,c,d^ Means without a common superscript are different (*p* < 0.05).

**Table 3:** Red blood cell profile of ETEC-challenged weaned piglets fed experimental diets.

Item ^1^	NC	PC	1% Glu	2% Glu	1% Asp	2% Asp	Carbadox	SEM	*p*-Value
**d** ** ** **0** ** **									
RBC, 10^6^/μL	7.59	7.76	7.57	7.29	7.76	8.01	8.09	0.282	0.426
HGB, g/dL	8.64 ^ab^	8.18 ^bc^	7.74 ^c^	7.80 ^c^	7.83 ^bc^	9.16 ^a^	8.20 ^bc^	0.318	0.012
HCT, %	33.67 ^ab^	33.49 ^abc^	30.91 ^c^	30.97 ^c^	32.39 ^bc^	35.19 ^a^	32.73 ^abc^	0.928	0.029
MCV, fL	44.49 ^a^	43.23 ^ab^	40.18 ^c^	43.28 ^ab^	41.90 ^bc^	44.11 ^a^	40.44 ^c^	0.923	0.001
MCH, pg	11.50 ^a^	10.60 ^ab^	10.30 ^b^	10.18 ^b^	10.13 ^b^	11.50 ^a^	10.16 ^b^	0.375	0.022
MCHC, g/dL	25.82	24.52	25.16	24.18	24.17	26.06	25.07	0.725	0.312
RDW, %	29.13	30.44	29.80	28.72	29.77	29.17	29.10	1.053	0.871
Platelets, 10^3^/μL	426.27	444.98	419.16	406.41	426.84	466.12	356.55	47.829	0.705
MPV, fL	8.42 ^ab^	7.81 ^abc^	7.72 ^bc^	7.40 ^c^	7.44 ^c^	8.64 ^a^	7.34 ^c^	0.312	0.021
Total protein, g/dL	5.54 ^b^	6.08 ^a^	5.45 ^b^	5.69 ^ab^	5.77 ^ab^	5.86 ^ab^	5.46 ^b^	0.176	0.041
**d** ** ** **2** ** ** **PI**									
RBC, 10^6^/μL	7.57	7.81	7.81	7.79	8.74	8.15	8.51	0.361	0.238
HGB, g/dL	8.61	8.26	8.16	8.19	8.96	9.34	8.47	0.438	0.427
HCT, %	32.96	32.91	32.23	31.84	35.61	34.41	33.24	1.479	0.575
MCV, fL	43.47 ^a^	42.14 ^ab^	40.51 ^bc^	40.73 ^bc^	41.06 ^abc^	42.26 ^ab^	39.21 ^c^	1.099	0.039
MCH, pg	11.93 ^a^	10.57 ^bc^	10.53 ^bc^	10.44 ^c^	10.33 ^c^	11.49 ^ab^	9.94 ^c^	0.368	0.003
MCHC, g/dL	27.49 ^a^	25.09 ^c^	26.03 ^abc^	25.57 ^bc^	25.13 ^c^	27.16 ^ab^	25.41 ^c^	0.588	0.028
RDW, %	26.54	28.74	28.31	26.91	27.49	26.71	27.27	0.799	0.215
Platelets, 10^3^/μL	369.99	420.27	386.87	379.99	415.13	387.42	372.99	61.914	0.991
MPV, fL	7.47	7.79	7.43	7.93	7.59	8.43	7.63	0.329	0.379
Total protein, g/dL	5.60 ^bc^	6.09 ^ab^	5.49 ^c^	5.97 ^abc^	6.45 ^a^	5.80 ^bc^	5.79 ^bc^	0.289	0.036
**d** ** ** **5** ** ** **PI**									
RBC, 10^6^/μL	8.03	8.19	7.41	8.35	8.02	7.93	8.08	0.340	0.636
HGB, g/dL	9.07	8.54	7.54	8.36	8.03	8.86	8.09	0.459	0.285
HCT, %	33.71	33.30	27.53	32.53	30.80	31.17	31.64	1.646	0.147
MCV, fL	42.00 ^a^	40.59 ^abc^	38.79 ^bcd^	38.77 ^bcd^	38.53 ^cd^	41.16 ^ab^	37.84 ^d^	0.952	0.018
MCH, pg	11.33	10.15	10.70	9.94	10.14	11.10	9.67	0.450	0.107
MCHC, g/dL	26.99 ^abc^	25.73 ^abc^	27.59 ^a^	25.40 ^bc^	25.12 ^c^	27.40 ^ab^	25.57 ^bc^	0.709	0.082
RDW, %	25.56	28.11	26.39	26.98	26.28	25.78	26.50	0.884	0.299
Platelets, 10^3^/μL	375.75	443.09	369.10	407.75	405.75	422.61	330.47	72.118	0.868
MPV, fL	8.02	7.68	7.79	7.99	7.55	8.84	7.39	0.403	0.130
Total protein, g/dL	5.45 ^c^	6.09 ^ab^	5.52 ^bc^	5.81 ^abc^	6.17 ^a^	5.50 ^bc^	5.57 ^bc^	0.208	0.085
**d** ** ** **14** ** ** **PI**									
RBC, 10^6^/μL	6.35	6.24	6.42	6.60	6.42	6.42	6.02	0.269	0.839
HGB, g/dL	8.01 ^a^	7.37 ^ab^	7.34 ^ab^	7.64 ^ab^	7.27 ^ab^	8.32 ^a^	6.60 ^b^	0.385	0.090
HCT, %	27.24	26.49	26.27	27.46	26.50	27.33	23.17	1.211	0.199
MCV, fL	43.04 ^a^	42.41 ^ab^	39.84 ^bc^	41.51 ^ab^	41.27 ^ab^	42.80 ^ab^	38.20 ^c^	1.034	0.023
MCH, pg	12.67 ^a^	11.80 ^ab^	11.92 ^ab^	11.54 ^ab^	11.33 ^b^	12.60 ^a^	10.89 ^b^	0.412	0.038
MCHC, g/dL	29.49 ^a^	27.64 ^b^	28.74 ^ab^	27.71 ^b^	27.47 ^b^	29.36 ^a^	28.51 ^ab^	0.542	0.052
RDW, %	25.94	26.78	25.35	26.13	26.07	26.01	25.08	0.987	0.614
Platelets, 10^3^/μL	345.43	305.00	319.71	345.14	299.43	322.57	278.29	46.124	0.932
MPV, fL	8.06 ^ab^	7.38 ^b^	7.40 ^b^	7.25 ^b^	7.29 ^b^	8.70 ^a^	7.25 ^b^	0.302	0.009
Total protein, g/dL	4.92	5.04	4.82	5.07	5.03	4.90	4.86	0.099	0.411

^1^ RBC = red blood cell; HGB = hemoglobin; HCT = hematocrit; MCV = mean corpuscular volume; MCH = mean corpuscular hemoglobin; MCHC = mean corpuscular hemoglobin concentration; RDW = red cell distribution width; MPV = mean platelet volume; PI = post-inoculation. Each least squares mean represents 7 replicates. ^a,b,c,d^ Means without a common superscript are different (*p* < 0.05).

**Table 4:** Significantly altered metabolites identified in serum, ileal mucosa, and ileal digesta of ETEC-challenged weaned piglets fed experimental diets.

Treatment Comparison ^1^	Fold Change ^2^	VIP ^3^ > 1.0	*p* < 0.05	FDR ^4^ < 0.2
***Serum, d 5 PI***				
**Car/NC**				
inositol-4-monophosphate	2.616	2.597	0.001	0.140
cholesterol	2.125	2.545	0.002	0.140
**1% Glu/NC**				
inosine	3.526	2.439	0.003	0.129
guanosine	3.505	2.419	0.003	0.129
tocopherol alpha-	3.043	2.503	0.002	0.129
inositol-4-monophosphate	2.498	2.800	<0.001	0.048
conduritol-beta-epoxide	2.410	2.749	<0.001	0.048
pinitol	2.351	2.627	0.001	0.070
cholesterol	2.079	2.365	0.004	0.150
**1% Asp/NC**				
inositol-4-monophosphate	2.773	2.560	0.001	0.109
cholesterol	2.336	2.596	0.001	0.109
***Ileal Mucosa, d 14 PI***				
**Car/NC**				
homovanillic acid	41.958	1.893	0.002	0.092
conduritol-beta-epoxide	17.958	1.761	0.005	0.128
pinitol	6.281	1.751	0.005	0.128
5-aminovaleric acid	5.917	2.027	<0.001	0.059
cis-sinapinic acid	4.699	1.827	0.003	0.106
pentitol	4.647	1.552	0.017	0.134
inosine	4.290	1.980	0.001	0.070
glucose	3.498	1.469	0.027	0.175
ribitol	3.299	1.669	0.009	0.128
xylose	3.208	1.600	0.013	0.128
nicotinic acid	2.818	1.503	0.023	0.156
sinapinic acid	2.761	1.662	0.009	0.128
nicotianamine	2.655	1.507	0.022	0.156
perseitol	2.645	1.656	0.010	0.128
tyrosol	2.611	1.442	0.030	0.186
vanillic acid	2.378	1.445	0.030	0.186
ferulic acid	2.287	1.640	0.011	0.128
cis-p-coumaric acid	2.259	1.425	0.033	0.186
indole-3-acetate	2.037	1.432	0.032	0.186
aminomalonic acid	0.477	1.389	0.039	0.198
glycine	0.365	1.431	0.032	0.186
**1% Asp/NC**				
pinitol	4.251	1.755	0.008	0.193
cis-sinapinic acid	4.124	1.853	0.004	0.149
panose	3.902	1.782	0.007	0.193
maltotriose	2.989	1.844	0.004	0.149
perseitol	2.729	1.982	0.002	0.086
beta-alanine	2.327	2.054	0.001	0.086
vanillic acid	2.224	1.977	0.002	0.086
**2% Asp/Car**				
inosine	0.408	2.038	0.005	0.199
indole-3-acetate	0.292	2.249	0.001	0.105
***Ileal Digesta, d 14 PI***				
**1% Glu/PC**				
cholesterol	0.460	2.024	0.003	0.104
palmitoleic acid	0.442	2.041	0.002	0.104
inositol-4-monophosphate	0.438	2.105	0.001	0.104
fructose-1-phosphate	0.396	1.851	0.008	0.194
oxalic acid	0.392	2.053	0.002	0.104
oleic acid	0.364	1.938	0.005	0.134
arachidonic acid	0.342	2.083	0.002	0.104
**1% Glu/NC**				
malonic acid	3.204	1.474	0.021	0.168
5-aminovaleric acid	2.314	1.605	0.010	0.120
valine	2.253	1.410	0.028	0.180
methionine	2.129	1.458	0.022	0.171
palmitoleic acid	0.485	1.467	0.021	0.168
threonic acid	0.485	1.792	0.002	0.055
lanosterol	0.476	1.563	0.012	0.128
2,5-dihydroxypyrazine	0.425	1.490	0.019	0.164
cholesterol	0.408	1.819	0.002	0.055
arachidonic acid	0.396	1.428	0.026	0.179
methanolphosphate	0.370	1.797	0.002	0.055
inositol-4-monophosphate	0.356	1.891	0.001	0.045
1-monopalmitin	0.355	1.449	0.023	0.176
zymosterol	0.351	1.812	0.002	0.055
icosenoic acid	0.338	1.607	0.009	0.120
oleic acid	0.338	1.484	0.020	0.166
pyrophosphate	0.329	1.964	<0.001	0.040
oxalic acid	0.310	2.061	<0.001	0.040
fructose-1-phosphate	0.299	1.401	0.030	0.185
**1% Glu/Car**				
2-hydroxybutanoic acid	0.483	1.893	0.004	0.179
N-acetylaspartic acid	0.460	1.878	0.004	0.179
inositol-4-monophosphate	0.391	1.834	0.006	0.198
oxalic acid	0.321	2.023	0.001	0.179
taurine	0.316	1.907	0.003	0.179
**1% Glu/2% Glu**				
oxalic acid	0.451	1.921	0.005	0.175
cholesterol	0.407	1.956	0.004	0.175
methanolphosphate	0.375	1.943	0.004	0.175
inositol-4-monophosphate	0.368	1.952	0.004	0.175
Pyrophosphate	0.351	2.274	<0.001	0.082

^1^ NC = negative control; PC = positive control; Glu = glutamate; Asp = aspartate; Car = carbadox. Each treatment included 7 replicates. ^2^ Fold change values greater than 2.0 indicate upregulation of metabolites between the treatment groups being compared, while values less than 1.0 indicate downregulation. The treatment listed before the slash (/) represents the numerator, and the treatment after the slash represents the denominator. ^3^ VIP = Variable Importance in Projection score from PLS-DA or similar. ^4^ FDR = False Discovery Rate, a statistical method used to correct for multiple comparisons.

**Table 5:** Pathway enrichment analysis of serum, ileal mucosa, and ileal digesta of ETEC-challenged weaned piglets fed experimental diets.

Treatment Comparison ^1^	Impact ^2^ > 0.1	*p* < 0.05	FDR ^3^ < 0.2	Total Metab. ^4^
***Serum, d 5 PI***				
**1% Asp vs. 2% Glu**				
Phenylalanine, tyrosine and tryptophan biosynthesis	1.000	0.017	0.152	4
Phenylalanine metabolism	0.357	0.020	0.152	8
Cysteine and methionine metabolism	0.168	0.013	0.152	33
Tyrosine metabolism	0.164	0.017	0.152	42
Tryptophan metabolism	0.143	0.011	0.152	41
**1% Asp vs. 2% Asp**				
Phenylalanine, tyrosine and tryptophan biosynthesis	1.000	0.024	0.150	4
Linoleic acid metabolism	1.000	0.043	0.178	5
Glycine, serine and threonine metabolism	0.536	0.017	0.150	33
Arachidonic acid metabolism	0.289	0.004	0.071	44
Tryptophan metabolism	0.143	0.002	0.054	41
***Ileal Mucosa, d 14 PI***				
**1% Glu vs. Car**				
Glycerolipid metabolism	0.330	0.018	0.156	16
Arachidonic acid metabolism	0.289	0.015	0.156	44
Tyrosine metabolism	0.165	0.020	0.156	42
Tryptophan metabolism	0.143	0.006	0.155	41
***Ileal Digesta, d 14 PI***				
**PC vs. Car**				
Taurine and hypotaurine metabolism	0.829	0.024	0.173	8
Galactose metabolism	0.252	0.023	0.173	27
Fructose and mannose metabolism	0.161	0.026	0.173	20
Inositol phosphate metabolism	0.124	0.018	0.173	30
**2% Asp vs. Car**				
Taurine and hypotaurine metabolism	0.829	0.002	0.041	8
Alanine, aspartate and glutamate metabolism	0.623	0.005	0.066	28
Arginine biosynthesis	0.406	0.009	0.068	14
Glyoxylate and dicarboxylate metabolism	0.259	0.002	0.041	31
**1% Glu vs. NC**				
Linoleic acid metabolism	1.000	0.037	0.164	5
Phenylalanine, tyrosine and tryptophan biosynthesis	1.000	0.047	0.164	4
Phenylalanine metabolism	0.357	0.047	0.164	8
Arachidonic acid metabolism	0.289	0.026	0.141	44
Cysteine and methionine metabolism	0.285	0.027	0.141	33
Steroid biosynthesis	0.168	0.001	0.044	41
Purine metabolism	0.123	0.012	0.097	70
Glycerophospholipid metabolism	0.118	0.013	0.097	36
**1% Glu vs. PC**				
Arachidonic acid metabolism	0.289	0.002	0.052	44
Steroid biosynthesis	0.168	0.003	0.052	41
Fructose and mannose metabolism	0.161	0.010	0.088	20
**1% Glu vs. Car**				
Linoleic acid metabolism	1.000	0.038	0.149	5
Taurine and hypotaurine metabolism	0.829	0.007	0.124	8
Alanine, aspartate and glutamate metabolism	0.623	0.007	0.124	28
Glycine, serine and threonine metabolism	0.536	0.028	0.149	33
Arachidonic acid metabolism	0.289	0.022	0.149	44
Glyoxylate and dicarboxylate metabolism	0.259	0.033	0.149	31
Pyrimidine metabolism	0.203	0.038	0.149	39
Steroid biosynthesis	0.168	0.038	0.149	41
Inositol phosphate metabolism	0.124	0.019	0.149	30
Purine metabolism	0.123	0.034	0.149	70
**1% Glu vs. 2% Glu**				
Arachidonic acid metabolism	0.289	0.007	0.131	44
Steroid biosynthesis	0.168	0.007	0.131	41
Glycerophospholipid metabolism	0.118	0.015	0.193	36

^1^ NC = negative control; PC = positive control; Glu = glutamate; Asp = aspartate; Car = carbadox. Each treatment included 7 replicates. ^2^ Pathway impact value represents the cumulative contribution of matched metabolite nodes, as determined by pathway topology analysis. ^3^ FDR = False Discovery Rate, a statistical method used to correct for multiple comparisons. ^4^ Total Metab. = Total Metabolites, the total number of metabolites associated with a given pathway.

## Data Availability

All data generated or analyzed during this study are available from the corresponding author upon reasonable request.

## Supplementary Materials

The following supporting information can be downloaded at https://www.mdpi.com/article/10.3390/metabo16040247/s1: Supplementary Figure S1: 2D PLS-DA score plot of d 5 PI serum metabolites revealed group separation patterns. Supplementary Figure S2: 2D PLS-DA score plot of d 5 PI serum metabolites revealed distinct pairwise treatment comparisons. Supplementary Figure S3: 2D PLS-DA score plot of d 14 PI ileal mucosa metabolites revealed group separation patterns. Supplementary Figure S4: 2D PLS-DA score plot of d 14 PI ileal mucosa metabolites revealed distinct pairwise treatment comparisons. Supplementary Figure S5: 2D PLS-DA score plot of d 14 PI ileal digesta metabolites revealed group separation patterns. Supplementary Figure S6: 2D PLS-DA score plot of d 14 PI ileal digesta metabolites revealed distinct pairwise treatment comparisons. Supplementary Table S1: Ingredient compositions of experimental diets, as-fed. Supplementary Table S2: Amino acid profiles of experimental diets. Supplementary Table S3: Sequences of oligonucleotide primers used for RT-qPCR assay. Supplementary Table S4: White blood cell profile of ETEC-challenged weaned pigs fed experimental diets.

## Abbreviations

The following abbreviations are used in this manuscript:

Asp	L-aspartate
ATP	Adenosine triphosphate
Baso	Basophil count
Car	Carbadox
cDNA	Complementary deoxyribonucleic acid
CFU	Colony-forming unit
CRP	C-reactive protein
CT	Cycle threshold
EDTA	Ethylenediaminetetraacetic acid
ELISA	Enzyme-linked immunosorbent assay
Eos	Eosinophil count
ETEC	Enterotoxigenic *Escherichia coli*
FDR	False discovery rate
GC/TOF-MS	Gas chromatography/time-of-flight mass spectrometry
Glu	L-glutamate
HCT	Hematocrit
HGB	Hemoglobin
Hp	Haptoglobin
IFN	Interferon
IL	Interleukin
LPS	Lipopolysaccharide
LT	Heat-labile toxin
Lym	Lymphocyte count
MCH	Mean corpuscular hemoglobin
MCHC	Mean corpuscular hemoglobin concentration
MCV	Mean corpuscular volume
Mono	Monocyte count
MPV	Mean platelet volume
mRNA	Messenger ribonucleic acid
NC	Negative control
Neu	Neutrophil count
Neu:Lym	Neutrophil-to-lymphocyte ratio
NF-κB	Nuclear factor kappa-light-chain-enhancer of activated B cells
NOD	Nucleotide-binding oligomerization domain
NTC	No template control
PBS	Phosphate-buffered saline
PC	Positive control
PI	Post-inoculation
PLS-DA	Partial least squares discriminant analysis
PWD	Post-weaning diarrhea
RT-qPCR	Reverse transcription quantitative polymerase chain reaction
RBC	Red blood cell count
RDW	Red cell distribution width
STa	Heat-stable toxin a
STb	Heat-stable toxin b
TCA	Tricarboxylic acid
TGF	Transforming growth factor
Th	T helper cell
TLR4	Toll-like receptor 4
TNF	Tumor necrosis factor
VIP	Variable importance in projection
WBC	White blood cell count

## References

[1] Moeser A.J., Pohl C.S., Rajput M. (2017). Weaning stress and gastrointestinal barrier development: Implications for lifelong gut health in pigs. Anim. Nutr..

[2] Fairbrother J.M., Nadeau É., Gyles C.L. (2005). *Escherichia coli* in postweaning diarrhea in pigs: An update on bacterial types, pathogenesis, and prevention strategies. Anim. Health Res. Rev..

[3] García V., Gambino M., Pedersen K., Haugegaard S., Olsen J.E., Herrero-Fresno A. (2020). F4- and F18-positive enterotoxigenic *Escherichia coli* isolates from diarrhea of postweaning pigs: Genomic characterization. Appl. Environ. Microbiol..

[4] Govindarajan D.K., Viswalingam N., Meganathan Y., Kandaswamy K. (2020). Adherence patterns of *Escherichia coli* in the intestine and its role in pathogenesis. Med. Microecol..

[5] Kim K., Song M., Liu Y., Ji P. (2022). Enterotoxigenic *Escherichia coli* infection of weaned pigs: Intestinal challenges and nutritional intervention to enhance disease resistance. Front. Immunol..

[6] He Y., Liu Y., Ji P. (2021). Metabolomic profile of weaned pigs challenged with *E. coli* and supplemented with carbadox or *Bacillus subtilis*. Metabolites.

[7] Monger X.C., Gilbert A.-A., Saucier L., Vincent A.T. (2021). Antibiotic resistance: From pig to meat. Antibiotics.

[8] Albernaz-Gonçalves R., Olmos Antillón G., Hötzel M.J. (2022). Linking animal welfare and antibiotic use in pig farming—A review. Animals.

[9] Barros M.M., Castro J., Araújo D., Campos A.M., Oliveira R., Silva S., Outor-Monteiro D., Almeida C. (2023). Swine colibacillosis: Global epidemiologic and antimicrobial scenario. Antibiotics.

[10] Wu G. (2013). Functional amino acids in nutrition and health. Amino Acids.

[11] Hou Y., Wu G. (2018). L-glutamate nutrition and metabolism in swine. Amino Acids.

[12] Pi D., Liu Y., Shi H., Li S., Odle J., Lin X., Zhu H., Chen F., Hou Y., Leng W. (2014). Dietary supplementation of aspartate enhances intestinal integrity and energy status in weanling piglets after lipopolysaccharide challenge. J. Nutr. Biochem..

[13] Rezaei R., Knabe D.A., Tekwe C.D., Dahanayaka S., Ficken M.D., Fielder S.E., Eide S.J., Lovering S.L., Wu G. (2013). Dietary supplementation with monosodium glutamate is safe and improves growth performance in postweaning pigs. Amino Acids.

[14] Wu G. (2009). Amino acids: Metabolism, functions, and nutrition. Amino Acids.

[15] Stegink L.D. (1976). Absorption, utilization, and safety of aspartic acid. J. Toxicol. Environ. Health.

[16] Xue H., Field C.J. (2011). New role of glutamate as an immunoregulator via glutamate receptors and transporters. Front. Biosci. (Schol. Ed.).

[17] Wang H., Liu Y., Shi H., Wang X., Zhu H., Pi D., Leng W., Li S. (2017). Aspartate attenuates intestinal injury and inhibits TLR4 and NODs/NF-κB and p38 signaling in weaned pigs after LPS challenge. Eur. J. Nutr..

[18] Duan J., Yin J., Ren W., Liu T., Cui Z., Huang X., Wu L., Kim S.W., Liu G., Wu X. (2016). Dietary supplementation with L-glutamate and L-aspartate alleviates oxidative stress in weaned piglets challenged with hydrogen peroxide. Amino Acids.

[19] Tang X., Xiong K., Fang R., Li M. (2022). Weaning stress and intestinal health of piglets: A review. Front. Immunol..

[20] Brosnan J.T., Brosnan M.E. (2013). Glutamate: A truly functional amino acid. Amino Acids.

[21] Wongchanla S., Dixit K., Park S., Kim K., Sun S., Marco M., Palomares S.B., Mejia-Caballero A., Mohan S., Li X. (2025). Effects of dietary L-glutamate and L-aspartate supplementation on growth performance, severity of diarrhea, intestinal barrier integrity, and fecal microbiota of weaned piglets challenged with F18 enterotoxigenic *Escherichia coli*. J. Anim. Sci. Biotechnol..

[22] National Research Council (U.S.) (2012). Nutrient Requirements of Swine.

[23] Kim K., He Y., Xiong X., Ehrlich A., Li X., Raybould H., Atwill E.R., Maga E.A., Jørgensen J., Liu Y. (2019). Dietary supplementation of *Bacillus subtilis* influenced intestinal health of weaned pigs experimentally infected with a pathogenic *E. coli*. J. Anim. Sci. Biotechnol..

[24] Liu Y., Song M., Che T.M., Almeida J.A.S., Lee J.J., Bravo D., Maddox C.W., Pettigrew J.E. (2013). Dietary plant extracts alleviate diarrhea and alter immune responses of weaned pigs experimentally infected with a pathogenic *Escherichia coli*. J. Anim. Sci..

[25] Fiehn O., Wohlgemuth G., Scholz M., Kind T., Lee D.Y., Lu Y., Moon S., Nikolau B. (2008). Quality control for plant metabolomics: Reporting MSI-compliant studies. Plant J..

[26] Gresse R., Chaucheyras-Durand F., Fleury M.A., Van de Wiele T., Forano E., Blanquet-Diot S. (2017). Gut microbiota dysbiosis in postweaning piglets: Understanding the keys to health. Trends Microbiol..

[27] Burrin D.G., Stoll B. (2009). Metabolic fate and function of dietary glutamate in the gut. Am. J. Clin. Nutr..

[28] Blachier F., Boutry C., Bos C., Tomé D. (2009). Metabolism and functions of L-glutamate in the epithelial cells of the small and large intestines. Am. J. Clin. Nutr..

[29] Qin Q., Xu X., Wang X., Wu H., Zhu H., Hou Y., Dai B., Liu X., Liu Y. (2018). Glutamate alleviates intestinal injury, maintains mTOR and suppresses TLR4 and NOD signaling pathways in weanling pigs challenged with lipopolysaccharide. Sci. Rep..

[30] Ganor Y., Levite M., Levite M. (2012). Glutamate in the immune system: Glutamate receptors in immune cells, potent effects, endogenous production and involvement in disease. Nerve-Driven Immunity.

[31] Pacheco R., Gallart T., Lluis C., Franco R. (2007). Role of glutamate on T-cell mediated immunity. J. Neuroimmunol..

[32] Chany C., Cerutti I. (1986). Aspartate-assisted immune stimulation: Its importance in antitumor and antiviral protection. Int. J. Cancer.

[33] Wang H., Zheng X., Liu B., Xia Y., Xin Z., Deng B., He L., Deng J., Ren W. (2021). Aspartate metabolism facilitates IL-1β production in inflammatory macrophages. Front. Immunol..

[34] Al Laham N., Elyazji M., Al-Haddad R., Ridwan F. (2015). Possible hematological changes associated with acute gastroenteritis among kindergarten children in Gaza. Ann. Med. Health Sci. Res..

[35] Rhouma M., Fairbrother J.M., Beaudry F., Letellier A. (2017). Post-weaning diarrhea in pigs: Risk factors and non-colistin-based control strategies. Acta Vet. Scand..

[36] Geloo Z.S., Ershler W.B. (2007). Hematology and aging. Encyclopedia of Gerontology.

[37] Gupta A. (2017). Nutritional Anemia in Preschool Children.

[38] Lee W.-J., Cha S., Shin M., Islam M.A., Cho C., Yoo H.S. (2011). Induction of Th1 polarized immune responses by thiolated Eudragit-coated F4 and F18 fimbriae of enterotoxigenic *Escherichia coli*. Eur. J. Pharm. Biopharm..

[39] Luo Y., Xu J., Zhang C., Jiang C., Ma Y., He H., Wu Y., Devriendt B., Cox E., Zhang H. (2019). Toll-like receptor 5-mediated IL-17C expression in intestinal epithelial cells enhances epithelial host defense against F4+ ETEC infection. Vet. Res..

[40] D’Angelo J.A., Dehlink E., Platzer B., Dwyer P., Circu M.L., Garay J., Aw T.Y., Fiebiger E., Dickinson B.L. (2010). The cystine/glutamate antiporter regulates dendritic cell differentiation and antigen presentation. J. Immunol..

[41] Dröge W., Eck H.-P., Betzler M., Schlag P., Drings P., Ebert W. (1988). Plasma glutamate concentration and lymphocyte activity. J. Cancer Res. Clin. Oncol..

[42] Ren W., Yin J., Gao W., Chen S., Duan J., Liu G., Li T., Li N., Peng Y., Yin Y. (2015). Metabolomics study of metabolic variations in enterotoxigenic *Escherichia coli*-infected piglets. RSC Adv..

[43] Gonzalez-Uarquin F., Rodehutscord M., Huber K. (2020). Myo-inositol: Its metabolism and potential implications for poultry nutrition—A review. Poult. Sci..

[44] Tu-Sekine B., Kim S.F. (2022). The inositol phosphate system—A coordinator of metabolic adaptability. Int. J. Mol. Sci..

[45] Zampelas A., Magriplis E. (2019). New insights into cholesterol functions: A friend or an enemy?. Nutrients.

[46] Tang Z., Ye W., Chen H., Kuang X., Guo J., Xiang M., Peng C., Chen X., Liu H. (2019). Role of purines in regulation of metabolic reprogramming. Purinergic Signal..

[47] Niki E., Noguchi N. (2021). Antioxidant action of vitamin E in vivo as assessed from its reaction products with multiple biological oxidants. Free Radic. Res..

[48] Zhou X., Liang J., Xiong X., Yin Y. (2024). Amino acids in piglet diarrhea: Effects, mechanisms and insights. Anim. Nutr..

[49] Jiang J., Yin L., Li J.-Y., Li Q., Shi D., Feng L., Liu Y., Jiang W.-D., Wu P., Zhao Y. (2017). Glutamate attenuates lipopolysaccharide-induced oxidative damage and mRNA expression changes of tight junction and defensin proteins, inflammatory and apoptosis response signaling molecules in the intestine of fish. Fish Shellfish Immunol..

[50] Harbige L.S. (2003). Fatty acids, the immune response, and autoimmunity: A question of n−6 essentiality and the balance between n−6 and n−3. Lipids.

[51] Cuervo L., McAlpine P.L., Olano C., Fernández J., Lombó F. (2024). Low-molecular-weight compounds produced by the intestinal microbiota and cardiovascular disease. Int. J. Mol. Sci..

[52] Selka A., Ndongou Moutombi F.J., Cormier M., Touaibia M. (2020). Phenethyl esters and amide of ferulic acid, hydroferulic acid, homovanillic acid, and vanillic acid: Synthesis, free radicals scavenging activity, and molecular modeling as potential cholinesterases inhibitors. Molbank.

[53] Deflaoui L., Mettouchi S., Setyaningsih W., Lovillo M.P., Barroso C.G., Tamendjari A. (2020). Effect of the harvesting period on the phenolic content and antioxidant activity of two Algerian olive cultivars. Riv. Ital. Sostanze Grasse.

[54] Yu M., Wen W., Yi X., Zhu W., Aa J., Wang G. (2022). Plasma metabolomics reveals diagnostic biomarkers and risk factors for esophageal squamous cell carcinoma. Front. Oncol..

[55] Tan X., Wang B., Zhou X., Liu C., Wang C., Bai J. (2024). Fecal fermentation behaviors of konjac glucomannan and its impacts on human gut microbiota. Food Chem. X.

[56] Obendorf R.L., Górecki R.J. (2012). Soluble carbohydrates in legume seeds. Seed Sci. Res..

[57] Razola-Díaz M.d.C., De Montijo-Prieto S., Aznar-Ramos M.J., Jiménez-Valera M., Ruiz-Bravo A., Verardo V., Gómez-Caravaca A.M. (2023). Effect of lactic acid bacteria fermentation on the polar compounds content with antioxidant and antidiabetic activity of avocado seed extracts. Fermentation.

[58] Culbertson J.Y., Kreider R.B., Greenwood M., Cooke M. (2010). Effects of beta-alanine on muscle carnosine and exercise performance: A review of the current literature. Nutrients.

[59] Yun K.-J., Koh D.-J., Kim S.-H., Park S.J., Ryu J.H., Kim D.-G., Lee J.-Y., Lee K.-T. (2008). Anti-inflammatory effects of sinapic acid through the suppression of inducible nitric oxide synthase, cyclooxygenase-2, and proinflammatory cytokine expressions via nuclear factor-κB inactivation. J. Agric. Food Chem..

[60] Prentki M., Madiraju S.R.M. (2008). Glycerolipid metabolism and signaling in health and disease. Endocr. Rev..

[61] Xu M., Wang X., Li Y., Geng X., Jia X., Zhang L., Yang H. (2021). Arachidonic acid metabolism controls macrophage alternative activation through regulating oxidative phosphorylation in a PPARγ-dependent manner. Front. Immunol..

[62] Wang J., Hao Y., Yang Y., Zhang Y., Xu C., Yang R. (2025). Gut microbiota-derived indole-3-acetic acid ameliorates precancerous inflammatory intestinal milieu to inhibit tumorigenesis through IL-35. J. Immunother. Cancer.

[63] Dodd D., Spitzer M.H., Van Treuren W., Merrill B.D., Hryckowian A.J., Higginbottom S.K., Le A., Cowan T.M., Nolan G.P., Fischbach M.A. (2017). A gut bacterial pathway metabolizes aromatic amino acids into nine circulating metabolites. Nature.

[64] Khalil B., Rosani A., Warrington S.J. (2024). Physiology, catecholamines. StatPearls.

[65] Funk C.D. (2001). Prostaglandins and leukotrienes: Advances in eicosanoid biology. Science.

[66] Henson C.P., Cleland W.W. (1964). Kinetic studies of glutamic oxaloacetic transaminase isozymes. Biochemistry.

[67] KEGG ENZYME: 1.13.11.34. https://www.genome.jp/entry/1.13.11.34.

[68] Meshram D., Bhardwaj K., Rathod C., Mahady G.B., Soni K.K. (2020). The role of leukotriene inhibitors in the management of chronic inflammatory diseases. Inflamm. Allergy Drug Targets.

[69] Lasunción M.A., Martín-Sánchez C., Canfrán-Duque A., Busto R. (2012). Post-lanosterol biosynthesis of cholesterol and cancer. Curr. Opin. Pharmacol..

[70] Sandhir R. (2014). Metabolic pathways|Lipid metabolism. Encyclopedia of Food Microbiology.

[71] Pearson A., Budin M., Brocks J.J. (2003). Phylogenetic and biochemical evidence for sterol synthesis in the bacterium *Gemmata obscuriglobus*. Proc. Natl. Acad. Sci. USA.

[72] Wang C., Peng Y., Zhang Y., Xu J., Jiang S., Wang L., Yin Y. (2023). The biological functions and metabolic pathways of valine in swine. J. Anim. Sci. Biotechnol..

[73] Yang Z., Htoo J.K., Liao S.F. (2020). Methionine nutrition in swine and related monogastric animals: Beyond protein biosynthesis. Anim. Feed Sci. Technol..

[74] Li Y., Han H., Yin J., Zheng J., Zhu X., Li T., Yin Y. (2018). Effects of glutamate and aspartate on growth performance, serum amino acids, and amino acid transporters in piglets. Food Agric. Immunol..

[75] Kim S., Bhandari R., Brearley C.A., Saiardi A. (2024). The inositol phosphate signalling network in physiology and disease. Trends Biochem. Sci..

[76] Suryavanshi M.V., Bhute S.S., Jadhav S.D., Bhatia M.S., Gune R.P., Shouche Y.S. (2016). Hyperoxaluria leads to dysbiosis and drives selective enrichment of oxalate-metabolizing bacterial species in recurrent kidney stone sufferers. Sci. Rep..

[77] Baltazar P., De Melo Junior A.F., Fonseca N.M., Lança M.B., Faria A., Sequeira C.O., Teixeira-Santos L., Monteiro E.C., Pinheiro L.C., Calado J. (2023). Oxalate (dys)metabolism: Person-to-person variability, kidney and cardiometabolic toxicity. Genes.

[78] Parthasarathy A., Cross P.J., Dobson R.C.J., Adams L.E., Savka M.A., Hudson A.O. (2018). A three-ring circus: Metabolism of the three proteogenic aromatic amino acids and their role in the health of plants and animals. Front. Mol. Biosci..

[79] Deters B.J., Saleem M. (2021). The role of glutamine in supporting gut health and neuropsychiatric factors. Food Sci. Hum. Wellness.

[80] Kim G., Weiss S.J., Levine R.L. (2014). Methionine oxidation and reduction in proteins. Biochim. Biophys. Acta Gen. Subj..

[81] KEGG PATHWAY: Glyoxylate and Dicarboxylate Metabolism—Fusarium Keratoplasticum. https://www.kegg.jp/pathway/fkr00630.

