# Supplementary material for: New Insights into Dietary L-Glutamate and L-Aspartate Modulation of Hematology, Immune Responses, and Metabolite Profiles in Enterotoxigenic Escherichia coli Challenged Piglets
Source: Metabolites. 2026 Apr 4;16(4):247. doi: 10.3390/metabo16040247 (PMC13117459; doi:10.3390/metabo16040247)
Supplement: Supplementary file 1 [file metabolites-16-00247-s001.zip › Supplementary Figure S4.pdf]

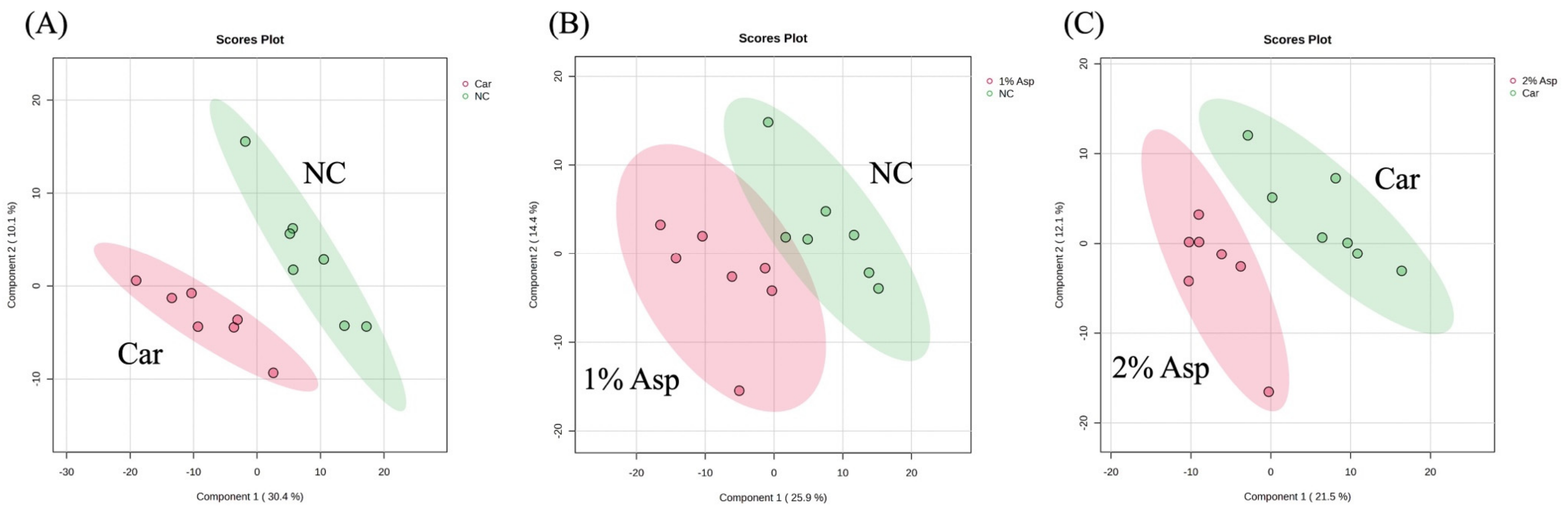

Supplementary Figure S4. 2D PLS-DA score plot of d 14 PI ileal mucosa metabolites revealed distinct pairwise treatment comparisons. Partial Least Squares Discriminant Analysis (PLS-DA) 2D score plot of the metabolites in ileal mucosa samples from d 14 PI showed clear separation between the NC and Car groups (A), NC and 1% Asp groups (B), and 2% Asp and Car groups (C). Each treatment included 7 replicates. NC = negative control; Car = carbadox; Asp = aspartate.
