# Supplementary material for: New Insights into Dietary L-Glutamate and L-Aspartate Modulation of Hematology, Immune Responses, and Metabolite Profiles in Enterotoxigenic Escherichia coli Challenged Piglets
Source: Metabolites. 2026 Apr 4;16(4):247. doi: 10.3390/metabo16040247 (PMC13117459; doi:10.3390/metabo16040247)
Supplement: Supplementary file 1 [file metabolites-16-00247-s001.zip › Supplementary Figure S5.pdf]

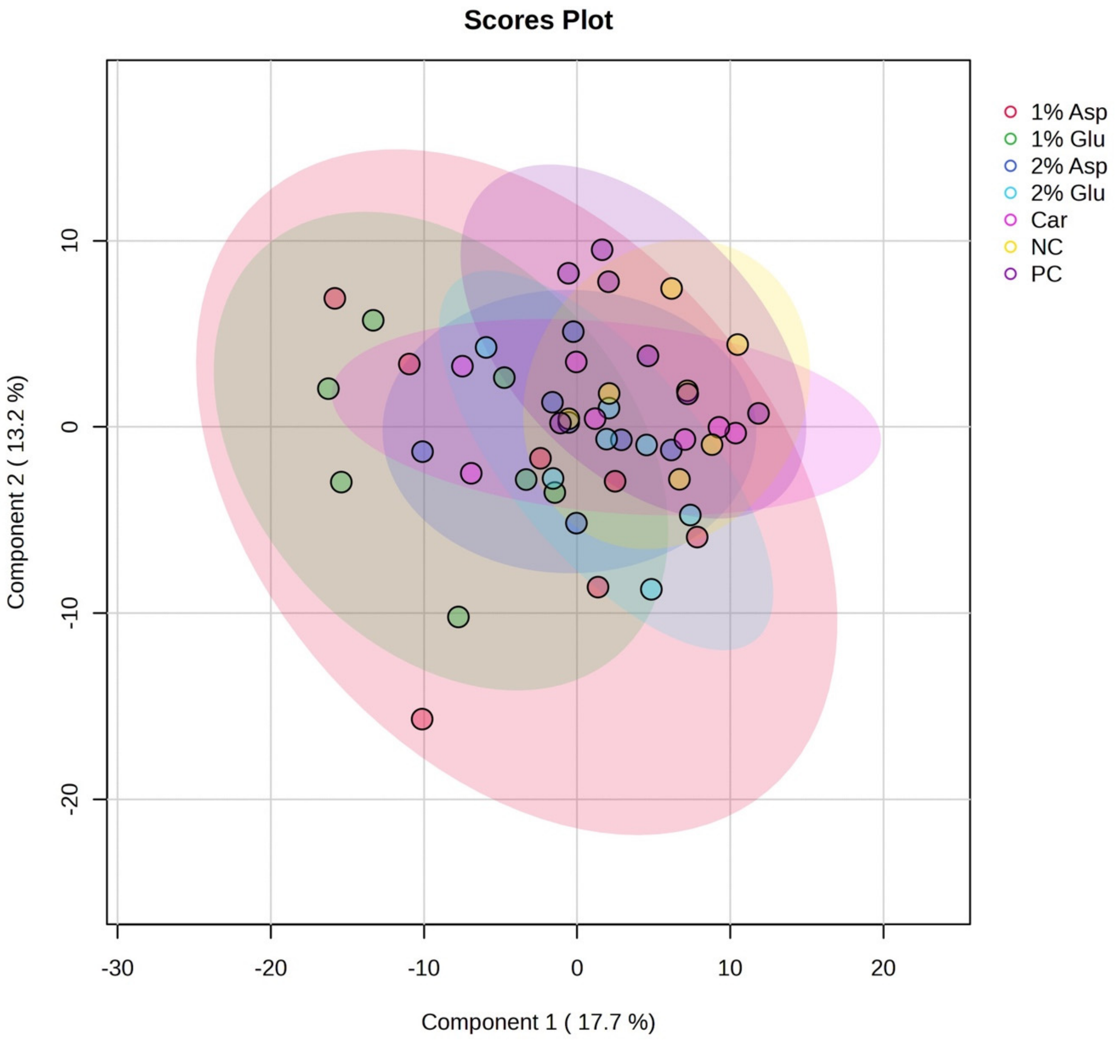


**Supplementary Figure S5**. **2D PLS-DA score plot of d 14 PI ileal digesta metabolites revealed group separation patterns.** Partial Least Squares Discriminant Analysis (PLS-DA) 2D score plot of the metabolites in ileal digesta samples from d 14 PI revealed trends in metabolic differentiation among the seven treatment groups. Component 1 explained 17.7% of the variance and Component 2 explained 13.2%. Model quality was evaluated using cross-validation parameters ($R^2$ and $Q^2$) generated in MetaboAnalyst 6.0 to assess goodness-of-fit and predictive ability. Each treatment included 7 replicates. NC = negative control; PC = positive control; Car = carbadox; Glu = glutamate; Asp = aspartate.
