# Supplementary material for: New Insights into Dietary L-Glutamate and L-Aspartate Modulation of Hematology, Immune Responses, and Metabolite Profiles in Enterotoxigenic Escherichia coli Challenged Piglets
Source: Metabolites. 2026 Apr 4;16(4):247. doi: 10.3390/metabo16040247 (PMC13117459; doi:10.3390/metabo16040247)
Supplement: Supplementary file 1 [file metabolites-16-00247-s001.zip › Supplementary Figure S6.pdf]

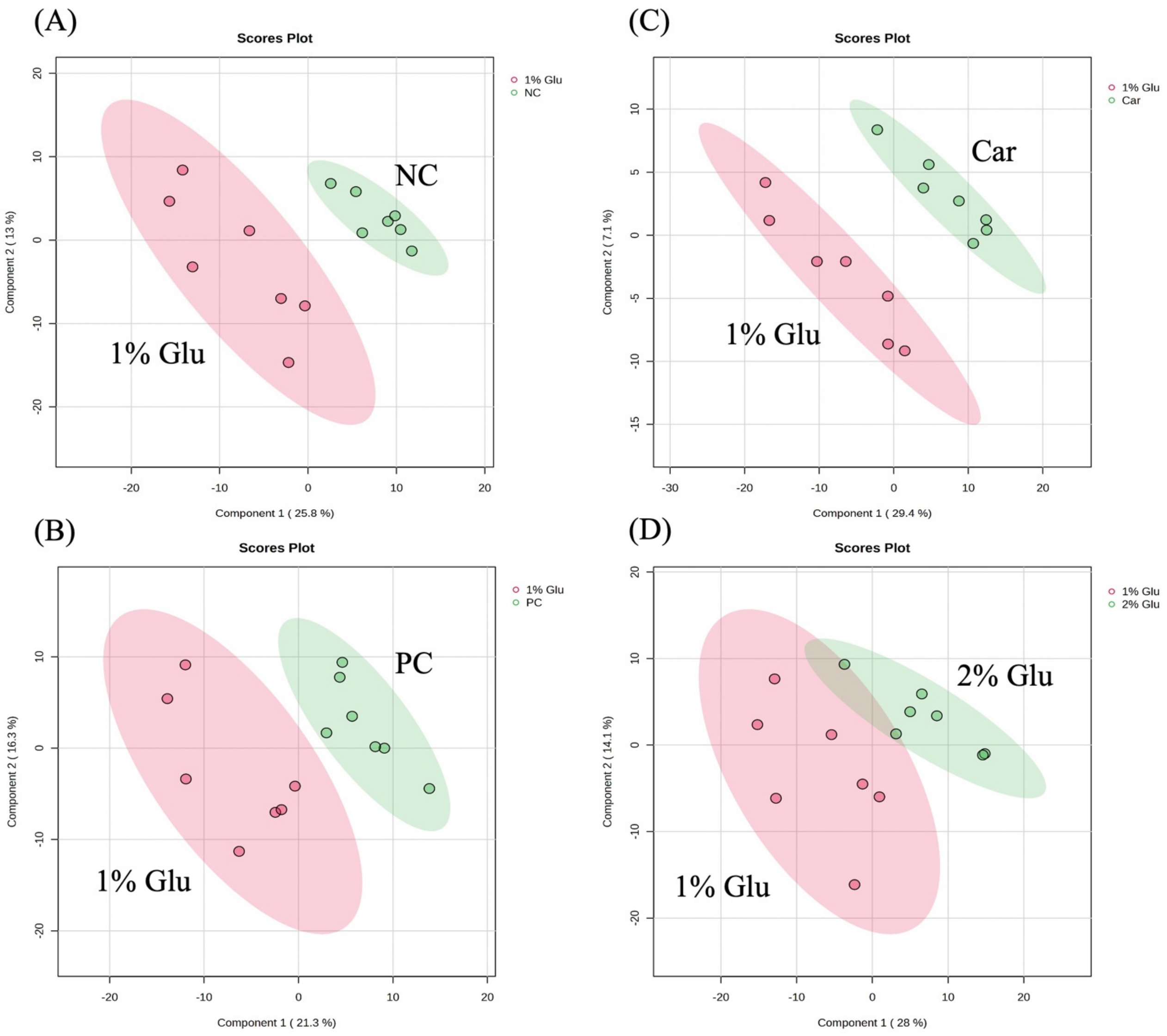

Supplementary Figure S6. 2D PLS-DA score plot of d 14 PI ileal digesta metabolites revealed distinct pairwise treatment comparisons. Partial Least Squares Discriminant Analysis (PLS-DA) 2D score plot of the metabolites in ileal digesta samples from d 14 PI showed clear separation between the 1% Glu and NC groups (A), 1% Glu and PC groups (B), 1% Glu and Car groups (C), and 1% Glu and 2% Glu groups (D). Each treatment included 7 replicates. NC = negative control; PC = positive control; Car = carbadox; Glu = glutamate.
