# Supplementary material for: New Insights into Dietary L-Glutamate and L-Aspartate Modulation of Hematology, Immune Responses, and Metabolite Profiles in Enterotoxigenic Escherichia coli Challenged Piglets
Source: Metabolites. 2026 Apr 4;16(4):247. doi: 10.3390/metabo16040247 (PMC13117459; doi:10.3390/metabo16040247)
Supplement: Supplementary file 1 [file metabolites-16-00247-s001.zip › Supplementary Table S1.pdf]

Supplementary Table S1. Ingredient compositions of experimental diets, as-fed[1]

Ingredient, %	Control, phase I	Control, phase II
Corn	44.70	54.29
Dried whey	15.00	10.00
Soybean meal	21.50	30.50
Fish meal	3.00	-
Lactose	6.00	-
Soy protein concentrate	5.00	-
Soybean oil	2.00	2.00
Limestone	0.98	1.00
Dicalcium phosphate	0.55	0.90
L-Lysine·HCl	0.34	0.39
DL-Methionine	0.14	0.12
L-Threonine	0.09	0.10
Salt	0.40	0.40
Vit-mineral premix[2]	0.30	0.30
Total	100.00	100.00
Analyzed nutrient, % as-is		
Dry matter	91.30	89.70
Crude protein	21.55	21.17
Acid detergent fiber	3.47	4.31
Neutral detergent fiber	7.94	9.06

[1]Diets were formulated based on NRC (2012) recommendations. Nutrient composition was determined using AOAC-approved methods, including Kjeldahl nitrogen analysis and Van Soest fiber procedures. In each phase, five additional diets were formulated accordingly.

[2]Provided by the United Animal Health (Sheridan, IN, USA). Provided the following quantities of vitamins and micro minerals per kilogram of complete diet: Vitamin A as retinyl acetate, 11,136 IU; vitamin D3 as cholecalciferol, 2,208 IU; vitamin E as DL-alpha tocopheryl acetate, 66 IU; vitamin K as menadione dimethylprimidinol bisulfite, 1.42 mg; thiamin as thiamine

mononitrate, 0.24 mg; riboflavin, 6.59 mg; pyridoxine as pyridoxine hydrochloride, 0.24 mg; vitamin B12, 0.03 mg; D-pantothenic acid as D-calcium pantothenate, 23.5 mg; niacin, 44.1 mg; folic acid, 1.59 mg; biotin, 0.44 mg; Cu, 20 mg as copper sulfate and copper chloride; Fe, 126 mg as ferrous sulfate; I, 1.26 mg as ethylenediamine dihydriodide; Mn, 60.2 mg as manganese sulfate; Se, 0.3 mg as sodium selenite and selenium yeast; and Zn, 125.1 mg as zinc sulfate.
