# Supplementary material for: New Insights into Dietary L-Glutamate and L-Aspartate Modulation of Hematology, Immune Responses, and Metabolite Profiles in Enterotoxigenic Escherichia coli Challenged Piglets
Source: Metabolites. 2026 Apr 4;16(4):247. doi: 10.3390/metabo16040247 (PMC13117459; doi:10.3390/metabo16040247)
Supplement: Supplementary file 1 [file metabolites-16-00247-s001.zip › Supplementary Table S2.pdf]

Supplementary Table S2. Amino acid profiles of experimental diets[1]

Item[2]	NC	PC	PC+1% Glu	PC+2%Glu	PC+1%Asp	PC+2%Asp	PC+carbadox
Phase I diet							
Arginine	1.18	1.28	1.29	1.21	1.24	1.13	1.24
Histidine	0.48	0.52	0.52	0.49	0.50	0.46	0.50
Isoleucine	0.94	1.01	1.01	0.96	0.98	0.90	0.99
Leucine	1.64	1.74	1.72	1.63	1.68	1.56	1.70
Lysine	1.43	1.52	1.48	1.51	1.46	1.37	1.47
Methionine	0.46	0.48	0.44	0.46	0.41	0.40	0.46
Phenylalanine	0.91	0.97	0.98	0.92	0.95	0.87	0.95
Threonine	0.83	0.89	0.88	0.84	0.83	0.80	0.85
Tryptophan	0.24	0.24	0.24	0.23	0.22	0.22	0.24
Valine	0.98	1.05	1.05	0.99	1.01	0.94	1.02
Alanine	0.98	1.03	1.01	0.96	1.01	0.92	1.00
Aspartic Acid	1.93	2.11	2.13	2.00	2.98	3.87	2.04
Cysteine	0.30	0.32	0.32	0.30	0.30	0.28	0.31
Glutamic Acid	3.35	3.60	4.47	5.52	3.53	3.25	3.53
Glycine	0.84	0.91	0.89	0.84	0.89	0.80	0.87
Proline	1.01	1.07	1.06	1.00	1.05	0.97	1.04
Serine	0.77	0.83	0.83	0.78	0.81	0.75	0.82
Tyrosine	0.59	0.63	0.64	0.61	0.63	0.58	0.63
Phase II diet							
Arginine	1.25	1.23	1.21	1.17	1.27	1.20	1.24
Histidine	0.50	0.50	0.49	0.48	0.52	0.49	0.50
Isoleucine	0.94	0.94	0.92	0.90	0.98	0.91	0.95
Leucine	1.66	1.66	1.63	1.58	1.71	1.60	1.66
Lysine	1.41	1.41	1.38	1.39	1.44	1.43	1.49
Methionine	0.43	0.39	0.39	0.38	0.38	0.41	0.42
Phenylalanine	0.95	0.94	0.93	0.90	0.98	0.92	0.95
Threonine	0.83	0.83	0.78	0.81	0.81	0.80	0.81
Tryptophan	0.22	0.23	0.23	0.23	0.24	0.23	0.22

Valine	0.98	0.97	0.96	0.94	1.01	0.96	0.98
Alanine	0.94	0.94	0.93	0.90	0.96	0.92	0.94
Aspartic Acid	1.99	1.97	1.94	1.91	2.98	3.79	2.00
Cysteine	0.31	0.30	0.32	0.29	0.32	0.29	0.33
Glutamic Acid	3.51	3.51	4.28	5.29	3.60	3.40	3.53
Glycine	0.78	0.78	0.77	0.75	0.81	0.77	0.79
Proline	1.03	1.03	1.01	0.98	1.06	1.00	1.03
Serine	0.82	0.80	0.79	0.77	0.84	0.78	0.81
Tyrosine	0.62	0.61	0.60	0.59	0.64	0.61	0.62

[1]Amino acid concentrations were determined according to standard AOAC International methods using cation-exchange high-performance liquid chromatography with post-column ninhydrin derivatization.

[2]W/W% = grams per 100 grams of sample. Results are expressed on an "as is" basis unless otherwise indicated.
