# Supplementary material for: New Insights into Dietary L-Glutamate and L-Aspartate Modulation of Hematology, Immune Responses, and Metabolite Profiles in Enterotoxigenic Escherichia coli Challenged Piglets
Source: Metabolites. 2026 Apr 4;16(4):247. doi: 10.3390/metabo16040247 (PMC13117459; doi:10.3390/metabo16040247)
Supplement: Supplementary file 1 [file metabolites-16-00247-s001.zip › Supplementary Table S3.pdf]

Supplementary Table S3. Sequences of oligonucleotide primers used for RT-qPCR assay

Gene[1]	Primer sequence (5'-3')[2]	Acc. No.[3]
18S rRNA	F: AGGAAAGCAGACATCGACCT<br>R: ACCTGGCTGTACTTCCCATC	AB117609.1
TNF-α	F: CACCACGCTCTTCTGCCTACT<br>R: GACGGGCTTATCTGAGGTTTGA	JF831365.1
IL-1β	F: GGTGACAACAATAATGACCTGTTATTTG<br>R: GCTCCCATT TCTCAGAGAACCA	NM_001302388.2
IL-6	F: GGGAAATGTCGAGGCTGTG<br>R: AGGGGTGGTGGCTTTGTCT	NM_214399
IFN-γ	F: GAGCCAAATTGTCTCCTTCTAC<br>R: CGAAGTCATTCAGTTTCCCAG	NM_213948.1
IL-12	F: CGTGCCTCGGGCAATTATA<br>R: CGCAGGTGAGGTCGCTAGTT	NM_213993.1
TGF-β1	F: GAAGCGCATCGAGGCCATTC<br>R: GGCTCCGGTTCGACACTTTC	NM_214015
IL-10	F: TCGGCCCAGTGAAGAGTTTC<br>R: GGAGTTCACGTGCTCCTTGA	JQ687536.1
IL-17A	F: CGGCTGGAGAAAGTGATGGT<br>R: CAGAAATGGGGCTGGGTCT	NM_001005729.1
IL-22	F: TTGACCAGTCCAACTTCCAGCAGC<br>R: GCAGCGCTCTCTCATATTGACTCC	XM_001926156.1
IL-23	F: TGTGGATCTACCAAGAGAAGAGG<br>R: AGGACTGACTGTTGTCCCTGA	GQ463150.1

[1]*18S rRNA* = 18S ribosomal ribonucleic acid; *TNF* = tumor necrosis factor; *IL* = interleukin; *IFN* = interferon; *TGF* = transforming growth factor.

[2]F = forward; R = reverse.

[3]Acc. No. = accession number in GenBank database.
