# Supplementary material for: New Insights into Dietary L-Glutamate and L-Aspartate Modulation of Hematology, Immune Responses, and Metabolite Profiles in Enterotoxigenic Escherichia coli Challenged Piglets
Source: Metabolites. 2026 Apr 4;16(4):247. doi: 10.3390/metabo16040247 (PMC13117459; doi:10.3390/metabo16040247)
Supplement: Supplementary file 1 [file metabolites-16-00247-s001.zip › Supplementary Table S4.pdf]

**Supplementary Table S4.** White blood cell profile of ETEC-challenged weaned pigs fed experimental diets

| Item[1] | NC | PC | 1% Glu | 2% Glu | 1% Asp | 2% Asp | Carbadox | SEM | *P*-value |
|---|---|---|---|---|---|---|---|---|---|
| **d 0** | | | | | | | | | |
| Neu, % | 44.68 | 48.73 | 41.68 | 43.90 | 46.87 | 48.26 | 41.86 | 3.240 | 0.479 |
| Lym, % | 42.69[ab] | 40.89[ab] | 48.27[a] | 47.17[a] | 43.69[ab] | 37.90[b] | 48.53[a] | 3.005 | **0.073** |
| Mono, % | 7.26 | 6.12 | 8.66 | 7.27 | 6.93 | 8.04 | 6.99 | 1.002 | 0.475 |
| Eos, % | 5.09[a] | 3.24[ab] | 1.31[b] | 1.41[b] | 2.27[b] | 5.38[a] | 2.14[b] | 0.933 | **0.007** |
| Baso, % | 0.10[b] | 0.12[b] | 0.17[b] | 0.11[b] | 0.15[b] | 0.14[b] | 0.39[a] | 0.072 | **0.073** |
| **d 2 PI** | | | | | | | | | |
| Neu, % | 46.96 | 46.24 | 46.38 | 50.51 | 47.09 | 52.58 | 44.13 | 2.946 | 0.465 |
| Lym, % | 42.03[abc] | 44.22[ab] | 43.23[ab] | 34.11[c] | 40.26[abc] | 36.36[bc] | 45.23[a] | 2.851 | **0.068** |
| Mono, % | 7.34 | 8.49 | 8.60 | 7.45 | 9.23 | 7.83 | 8.66 | 1.097 | 0.784 |
| Eos, % | 3.52 | 1.68 | 2.26 | 4.17 | 3.13 | 2.98 | 1.79 | 0.888 | 0.403 |
| Baso, % | 0.10 | 0.23 | 0.13 | 0.17 | 0.23 | 0.19 | 0.13 | 0.042 | 0.163 |
| **d 5 PI** | | | | | | | | | |
| Neu, % | 48.06[bc] | 53.87[ab] | 49.09[bc] | 57.39[a] | 52.52[abc] | 52.90[abc] | 46.97[c] | 2.632 | **0.035** |
| Lym, % | 41.00[ab] | 35.63[bc] | 39.78[ab] | 31.67[c] | 38.15[b] | 36.32[bc] | 44.18[a] | 2.095 | **0.003** |
| Mono, % | 8.68 | 7.09 | 8.63 | 5.36 | 7.26 | 7.51 | 8.10 | 0.868 | 0.122 |
| Eos, % | 2.24 | 1.31 | 2.23 | 2.27 | 1.61 | 1.31 | 0.40 | 0.539 | 0.164 |
| Baso, % | 0.15 | 0.16 | 0.15 | 0.18 | 0.16 | 0.27 | 0.21 | 0.042 | 0.273 |
| **d 14 PI** | | | | | | | | | |
| Neu, % | 51.79 | 52.34 | 47.17 | 53.88 | 54.38 | 52.24 | 44.03 | 3.612 | 0.353 |
| Lym, % | 40.33 | 40.89 | 46.36 | 38.70 | 38.71 | 35.58 | 49.03 | 3.513 | 0.136 |

| | | | | | | | | | |
|---|---|---|---|---|---|---|---|---|---|
| Mono, % | 6.90 | 5.49 | 4.85 | 6.12 | 5.35 | 7.28 | 6.32 | 0.989 | 0.458 |
| Eos, % | 0.84 | 0.48 | 1.07 | 0.65 | 1.30 | 1.54 | 0.41 | 0.318 | 0.127 |
| Baso, % | 0.09[b] | 0.19[a] | 0.25[a] | 0.16[ab] | 0.22[a] | 0.23[a] | 0.16[ab] | 0.036 | **0.025** |

[1]Neu = neutrophil; Lym = lymphocyte; Mono = monocyte; Eos = eosinophil; Baso = basophil; Each least squares mean represents 7 replicates. [a,b,c]Means without a common superscript are different ($P < 0.05$).
